# ANXA9 facilitates S100A4 and promotes breast cancer progression through modulating STAT3 pathway

**DOI:** 10.1038/s41419-024-06643-4

**Published:** 2024-04-12

**Authors:** Xiqian Zhou, Junyong Zhao, Tao Yan, Danrong Ye, Yuying Wang, Bai’an Zhou, Diya Liu, Xuehui Wang, Wenfang Zheng, Bowen Zheng, Fengyuan Qian, Yating Li, Dengfeng Li, Lin Fang

**Affiliations:** 1 Department of Breast and Thyroid Surgery, Shanghai Tenth People’s Hospital, Tongji University School of Medicine, Shanghai, China; 2 Institute of Breast Disease, School of Medicine, Tongji University, Shanghai, China

**Keywords:** Breast cancer, Oncogenes

## Abstract

Breast cancer has the highest global incidence and mortality rates among all cancer types. Abnormal expression of the Annexin family has been observed in different malignant tumors, including upregulated ANXA9 in breast cancer. We found highly expressed ANXA9 in metastatic breast cancer tissues, which is correlated with breast cancer progression. In vitro, the functional experiments indicated ANXA9 influenced breast cancer proliferation, motility, invasion, and apoptosis; in vivo, downregulation of ANXA9 suppressed breast cancer xenograft tumor growth and lung metastasis. Mechanically, on one side, we found that ANXA9 could mediate S100A4 and therefore regulate AKT/mTOR/STAT3 pathway to participate p53/Bcl-2 apoptosis; on the other side, we found ANXA9 transferred S100A4 from cells into the tumor microenvironment and mediated the excretion of cytokines IL-6, IL-8, CCL2, and CCL5 to participate angiogenesis via self- phosphorylation at site Ser2 and site Thr69. Our findings demonstrate significant involvement of ANXA9 in promoting breast cancer progression, thereby suggesting that therapeutic intervention via targeting ANXA9 may be effective in treating metastatic breast cancer.

## Introduction

Recent reports suggest that breast cancer (BC) accounts for approximately 30% of all female cancers [1]. It is estimated that 42,000 new BC cases were diagnosed in China in 2022, with approximately one-third expected to succumb to the metastatic disease [2]. Due to recent therapeutic advancements for BC, the five-year survival rate has been improved. It is estimated that approximately 95% of BC patients with stage I disease achieved a minimal five-year survival, whereas this survival rate declines sharply to a mere 28% for those diagnosed with stage IV disease [3]. Therefore, metastatic BC (MBC) remains an extremely burdensome and challenging cancer to treat.

To explore potential targeted therapies for MBC, we searched the ONCOMINE database with the term “metastasis” for BC. We found that Annexin 9 (ANXA9) was highly expressed in the MBC group compared to the non-metastasis group (Supplement Table 1). The ANXA9, a member of the Annexin family, is a calcium-dependent phospholipid-binding protein and can cooperatively bind anionic phospholipids and extracellular matrix proteins (https://www.ncbi.nlm.nih.gov/gene/8416). Several studies revealed that high expression of ANXA9 could promote cancer cellular proliferation and migration via activating the transforming growth factor-β (TGF-β) pathway in gastric cancer [4]. ANXA9 has been reported to be associated with gastric cancer grade, tumor protein 53 (TP53) mutation, histological subtype, and immune cellular infiltration [5]. In BC, ANXA9 was reported to be associated with estrogen receptor 1 (ER1) and can be upregulated by estrogen receptor beta (ERβ), which correlates with BC bone metastasis [6, 7], rendering it a potentially valuable target for BC [8].

However, research into the specific molecular mechanisms of ANXA9 in BC remains limited. Since ANXA9 is one of the sub-molecules within ANXAs, we searched several databases on ANXAs to understand their potential mechanisms. It was reported that annexin A2 (ANXA2) could regulate S100 calcium-binding protein A10 (S100A10) in an active calcium-bound form [9]. Pally et al. reported that S100A4 contributes to BC progress through extracellular matrix [10]. Another study demonstrated that FGF2/FGFR1 mediate triple-negative breast cancer (TNBC) cells to upregulate and secret S100A4 to adapt vascular endothelial cells and trigger motility of cancer-associated fibroblasts [11].

In our study, ANXA9 was found highly expressed in BC tissues, particularly in MBC. The ANXA9 protein may regulate S100A4 to mediate BC progression, which may lead to the development of BC targeted therapies.

## Methods and materials

### Specimen collection

The inclusion criteria for acquiring specimens from BC patients at the Tenth Hospital in Shanghai, China, included that the individuals were surgical candidates who had not undergone prior radiotherapy or chemotherapy. Informed consent was obtained from all participants, and the study was approved by the Ethics Committee of Shanghai Tenth Hospital (Ethics approval number: 23KN178).

### RNA extraction and real-time quantitative polymerase chain reaction (RT-qPCR)

We extracted RNA from the specimen and BC cells with an EZ-press RNA Purification Kit (EZBioscience, New Mexico, USA). Then, the total RNA was reversed to cDNA by using ABScript III RT Master Mix with gDNA remover (Abclonal, Wuhan, China). We then used 2× Universal SYBR Green Fast qPCR Mix (Abclonal, Wuhan, China) to prepare the 10 μL system and ABI QuanStudio Dx (Thermo Fisher Scientific, Massachusetts, USA) was used to conduct RT-qPCR program. The relative expression levels of each gene were compared by the method of 2^-△△ct^. The sequences of primers could be found in Supplement data 2.

### Cell culture, transfection, and stable cell line establishment

Human TNBC cells (MDA-MB-231 and BT549) and luminal BC cell line (MCF-7) were purchased from the Chinese Academy of Science at Shanghai (Shanghai, China). The culture media was Dulbecco’s modified Eagle’s medium (DMEM, WISENT, Canada) supplied with 10% fetal bovine serum (FBS, Absin, Shanghai, China) and 1% penicillin-streptomycin (PS, Beyotime, Shanghai, China).

Small interfering RNAs (siRNAs) and plasmids were designed and synthesized by Generay Biotech (Shanghai, China). Lipofectamine 8000 (Lipo8000, Beyotime, Shanghai, China) were used for transient transfection and the concentration of siRNAs were 100 nM and plasmids were 2 µg/ml. The short hairpin RNAs (shRNAs) plasmids were designed and synthesized by GentleGen (Suzhou, China). The LentiFit transfection kit (Hanbio, Shanghai, China) was applied to establish stable cell lines. The sequences of primers could be found in Supplement data 2.

### Proliferation assays

Transfected BC cells were re-suspended and seeded in 96-well plates for 3-(4,5-dimethylthiazolyl-2)-2,5-diphenyltetrazolium bromide (MTT) assay. The degree of proliferation was quantified by measuring the absorbance of MTT (Beyotime, Shanghai, China) at 490 nm OD. Moreover, the cells were also seeded in six-well plates for colony formation assay. These six-well plates were fixed with 4% paraformaldehyde (PFA, Servicebio, Wuhan, China) and dyed with 0.1% crystal violet (Yeasen, Shanghai, China) after culturing for 7-10 days.

### Wound healing assay

Transfected BC cells were re-seeded in six-well plates for the wound healing assay. Every well of the plate was scratched by a 200 μL sterile tip when the confluence of BC cells reached 90%. Then, the culture medium was changed to DMEM supplied with 2% FBS. Photos of each wound were taken at 0 h, 24 h, and 48 h after changing the culture medium. The scratch width was then used to calculate migration speed.

### Migration and Invasion assay

Transfected BC cells were re-suspended in DMEM without FBS, then single cell suspension was seeded in each upper chamber of the 24-well transwell cup (Corning, NY, USA) without or with matrix to test the ability of migration or invasion of BC cells. Stattic (10 μM) [12] was used to inhibit the STAT3 phosphoylation and Cardamonin (20 μM) [13] was used to inhibit AKT/mTOR/STAT3 signal pathway. The suspension was removed after 16 h of culture, and the bottom of each cup was washed with phosphate buffer saline (PBS, Servicebio, Wuhan, China), fixed with 4% PFA, and stained with 0.1% crystal violet. Photos of the bottoms were captured after they were air-dried.

### Flow cytometry

The flow cytometry was performed to quantify the cellular apoptosis rate. Transfected BC cells were digested by trypsin without EDTA (Beyotime, Shanghai, China). Both cell culture medium and adherent cells were collected and double stained with Annexin V-FITC and PI (Beyotime, Shanghai, China). The early apoptosis rate of BC cells was detected and measured with the BD FASC Canto II flow cytometry (BD Biosciences, San Jose, CA, USA).

### Proteasome degradation assay

Transfected BC cells were treated with cycloheximide (CHX, 25 μg/mL, MCE, New Jersey, USA; Cat# HY-12320) for 15, 30, 60 and 120 min. The cells were also treated with MG132 (10 μM, MCE, New Jersey, USA; Cat# HY-13259) for 4 h. All the samples were collected and extracted separately with RIPA (Servicebio, Wuhan, China) for subsequent western blot assay.

### Co-immunoprecipitation (Co-IP)

For Co-IP, original BC cells were extracted with cell lysis buffer for IP (Beyotime, Shanghai, China). 1000 μg protein of each sample was diluted in 500 μL IP lysis buffer. Specific antibodies (3 μg, ANXA9(Abcam, Cambridge, UK; Cat# ab166621), S100A4(Abcam, Cambridge, UK; Cat# ab197896) and HA-tag (Abclonal, Wuhan, China; Cat# AE008)) were incubated with the cell lysates and blended gently overnight at 4 °C. The homologous antibody with non-specific immunity (Rabbit pAb Control IgG, Abclonal, Wuhan, China, Cat# AC005; or Mouse pAb Control IgG, Abclonal, Wuhan, China, Cat# AC011) was used as control. Then 5 μL Protein A and 5 μL Protein G (absin, Shanghai, China) were added into each sample and blended gently for 4 h at 4 °C. After washing thrice in wash buffer (absin, Shanghai, China), captured proteins were eluted and denatured in 40 μL 1X SDS-PAGE Sample Loading Buffer (Beyotime, Shanghai, China) at 95 °C for 5 min. Finally, we used western blots to detect the binding proteins in these samples.

### Western blot analysis

Protein extracted as described in our previous manuscript [14]. Every sample (30 μg) was separated by using vertical electrophoresis with SDS polyacrylamide gels (Yeasen, Shanghai, China) with densities from 6% to 12% and electro-transferred to 0.2 μm nitrocellulose (NC) filter membranes (Cytiva, Massachusetts, USA). Then, 5% skim milk or bovine serum albumin (BSA, Yeasen, Shanghai, China) was used for blocking, and the membranes were incubated with specific primary antibodies at 4 °C overnight. The membranes were then incubated with commonly used secondary antibodies at room temperature for 1 h. Membranes were washed with TBST (TBS supplied with 0.1% Tween-20, Sangon Biotech, Shanghai, China). Subsequently, these membranes were detected with infrared thermography technology by using an Odyssey scanning system (LI-COR Biosciences, Lincoln, NE, USA). The information of antibodies could be found in Supplement data 2.

### Enzyme-linked immunosorbent assay (ELISA)

BC cells were seeded and transfected in six-well plates; the medium was changed 24 h after the transfection and cultured for another two days. Then, the cell culture was collected respectively, centrifuged at 1000 g for 20 min, and the supernatant was separated. After that, target proteins in the supernatant were detected with specific ELISA kits (Shanghai Ruifan Biological Technology Co, Ltd, Shanghai, China) according to the manufacturers’ protocol. SpectraMax iD5 (Molecular Devices, California, USA) was used to measure the OD value at 450 nm wavelength. All the ELISA kits (S100A4, IL-6, IL-8, CCL-2, CCL-5, and VEGF) were purchased from Shanghai Ruifan Biological Technology Co, Ltd (Shanghai, China).

### In vivo experiments

As previously stated, we performed in vivo experiments using constructed ANXA9 or S100A4 knocked-out (KO) MDA-MB-231 and BT549 cells and their negative control. The Medical Science Innovation Center of Shanghai Tenth People’s Hospital helped purchase the 4–6 weeks-old female nude mice (JiHui, Shanghai, China) and provided a breeding environment that was specific pathogen-free (SPF). This project obtained the permit of the Animal Ethics Committee of Shanghai Tenth Hospital (Ethics number: SHDSYY-2023-0600). For xenograft experiments, 1 × 10^6^ cells were injected under the armpit of every female mouse. The nude mice were randomly allocated into groups, each consisting of six mice. The weight of the mice and the size of the tumor were measured every two days, while the volume of the tumor was calculated as in our previous study [14]. We terminated the xenograft experiment when the tumor volume of either group approached 1 cm^3^. The tumor was partially fixed with 4% PFA (Servicebio, Wuhan, China), and partially rapidly frozen with liquid nitrogen. For lung metastasis experiments, 5 × 10^5^ cells were rapidly injected into the tail vein. The weight of the mice was measured every two days. We terminated the lung metastasis experiment when either group of mice showed significant weight loss or shortness of breath. For subsequent experiments, they were fixed in 4% PFA (Servicebio, Wuhan, China).

### Hematoxylin-eosin (HE) and Immunohistochemical staining (IHC)

Fixed tumors and tissues were embedded with paraffin wax. We were assisted by Servicebio (Wuhan, China) in preparing paraffin sections for HE staining and IHC staining. The information of antibodies could be found in Supplement data 2.

### Statistical analysis

SPSS v20.0 (Illinois, USA), Graphpad Prism v5.0 software (California, USA) and R v4.3.1 software (Auckland, New Zealand) were used to perform the data analysis. Each data set consisted of three duplicates from at least three independent experiments. All data were presented as mean and standard deviation, except for the in vivo experiments, which were presented as mean ± standard error of the mean. Student’s t-test or two-way analysis of variance test was used for comparisons, and the results were considered significant when the p-value was less than 0.05.

## Results

### ANXA9 is highly expressed in breast cancer

**Fig. 1:**
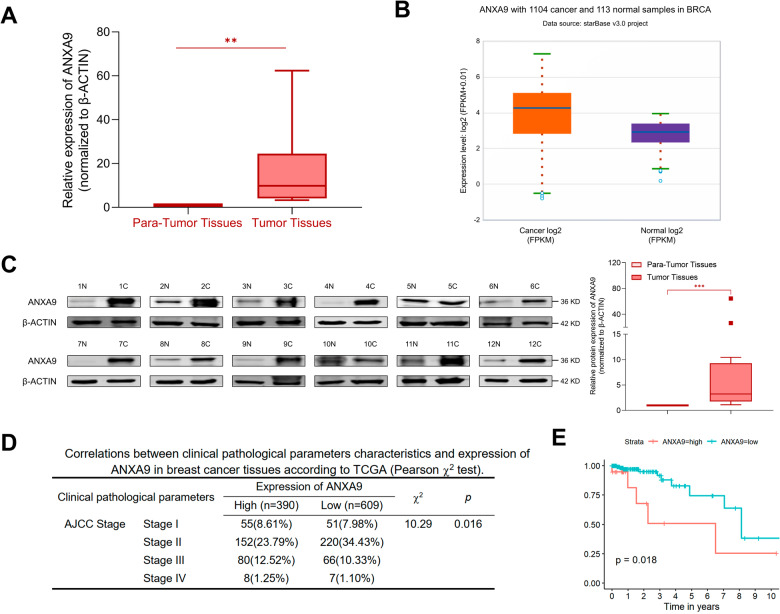
ANXA9 is highly expressed in breast cancer. **A**, **B** ANXA9 is highly expressed in BC tissues; **C** BC tissues contained higher expression of ANXA9 than para-cancer tissues assessed by Western blot; **D** The clinical pathological data from TCGA showed high expression of ANXA9 was connected with AJCC stage of BC (χ^2^ = 10.29, *p* = 0.016); **E** Bioinformatics analysis of TCGA showed high levels of ANXA9 were associated with poor outcome among BC patients with metastases (*n* = 184, *p* = 0.018). **p < 0.01, ***p < *0.001*. All of the experiments were replicated for three times.

As demonstrated in Fig. 1A, ANXA9 was highly expressed in our BC specimens. The ENCORI database (https://starbase.sysu.edu.cn/panCancer.php) also showed highly expressed ANXA9 in BC tissues (Fig. 1B). Besides, the protein expression level of ANXA9 is higher in clinical BC tissues (Fig. 1C). Furthermore, the high expression of ANXA9 was related to AJCC stage of BC (χ^2^ = 10.29, *p* = 0.016) according to the clinical pathological data from TCGA (Fig. 1D). High expression of ANXA9 was associated with poor prognosis in TCGA database (Fig.1E). All these data suggested that ANXA9 is highly expressed in BC tissues and associated with poor prognosis.

### ANXA9 induces breast cancer progression

**Fig. 2:**
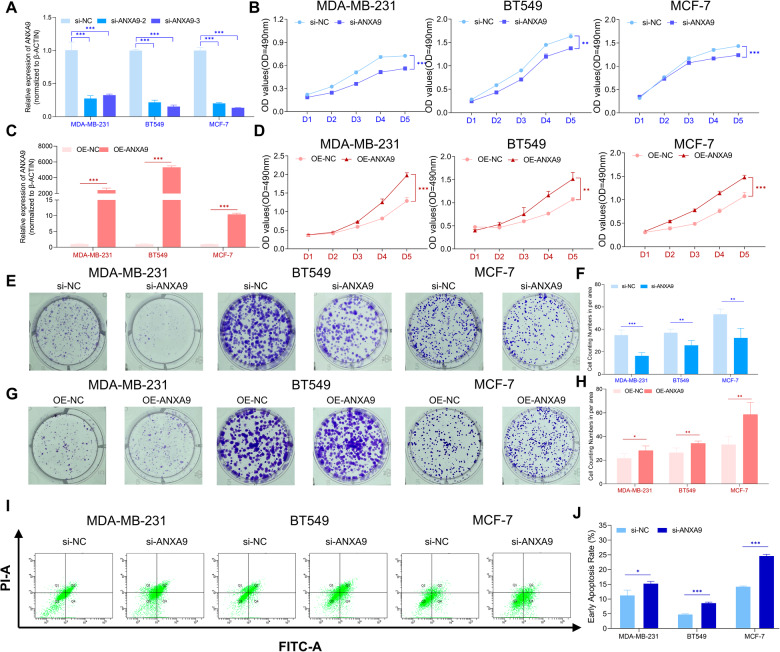
Biological functions of ANXA9 in breast cancer cells. **A** The transfection efficiency of small interfering of ANXA9 in BC cells; **B** ANXA9 suppressed BC cellular proliferation via MTT assays; **C** The transfection efficiency of plasmids of ANXA9 in BC cells; **D** ANXA9 enhanced BC cellular proliferation via MTT assays; **E**–**H** ANXA9 affected BC cellular colony-forming ability; **I**, **J** Downregulation of ANXA9 induced early apoptosis in BC cells. *p < 0.05, **p < 0.01, ***p < *0.001*. All of the experiments were replicated for three times.

As shown in Fig. 2A, C, two of siRNAs or OE-plasmid could significantly decrease or increase ANXA9 expression in BC cells. The siRNA of ANXA9 significantly suppressed BC cell proliferation (Fig. 2B) and ANXA9 overexpression increased BC cell proliferation in MTT assays (Fig. 2D). The colony formation assay also confirmed that ANXA9 downregulation decreased colony formation abilities (Fig. 2E, F) and ANXA9 overexpression increased colony formation (Fig. 2G, H). In addition, as illustrated in Fig. 2I, J, ANXA9 downregulation increased early apoptosis in MDA-MB-231, BT549, and MCF-7 cells.

**Fig. 3:**
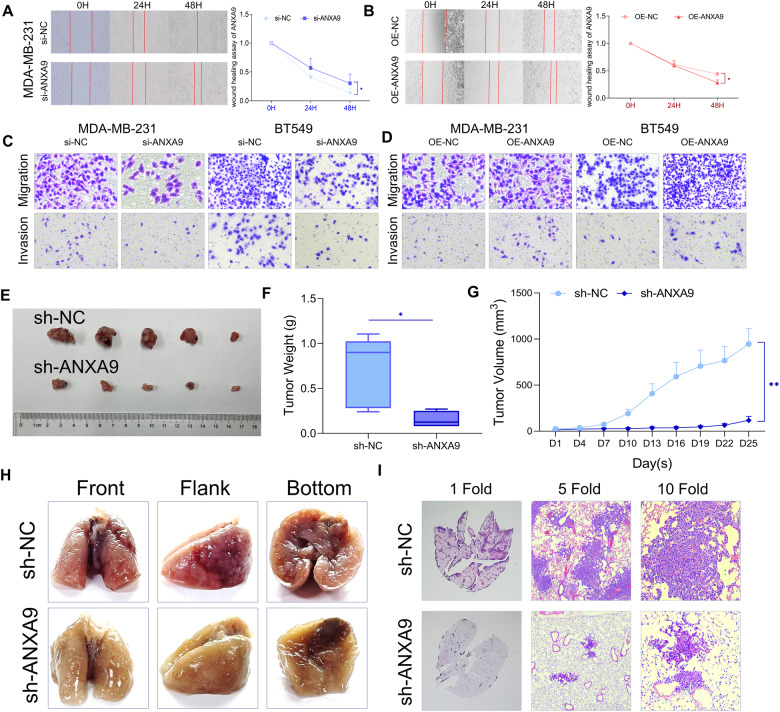
ANXA9 affects the progression of breast cancer cells. **A**, **B** ANXA9 affected MDA-MB-231 cellular scratch healing ability; **C**, **D** ANXA9 affected BC cellular motility. **E**–**G** sh-ANXA9 inhibits the tumorigenic ability of BT549 cells in nude mice; **H**, **I** The sh-ANXA9 group decreased the number of pulmonary metastatic foci in mice lung metastasis model. *p < 0.05, **p < *0.01*. All of the experiments were replicated three times.

The wound-healing results revealed that decreased ANXA9 expression suppressed the motility of MDA-MB-231 cells, and upregulation increased that (Fig. 3A, B). The transwell assay also confirmed that ANXA9 downregulation suppressed the motility of MDA-MB-231 and BT549 cells, and ANXA9 upregulation increased that (Fig. 3C, D). Moreover, as presented in Fig. 3E–G, ANXA9 knock-down significantly suppressed xenograft tumor growth. And the lung metastasis model found that fewer lesions in the sh-ANXA9 groups (Fig. 3H, I). All these findings suggest that ANXA9 contributes to BC progression and plays a vital function in BC metastasis.

### ANXA9 interacts with S100A4 in breast cancer

**Fig. 4:**
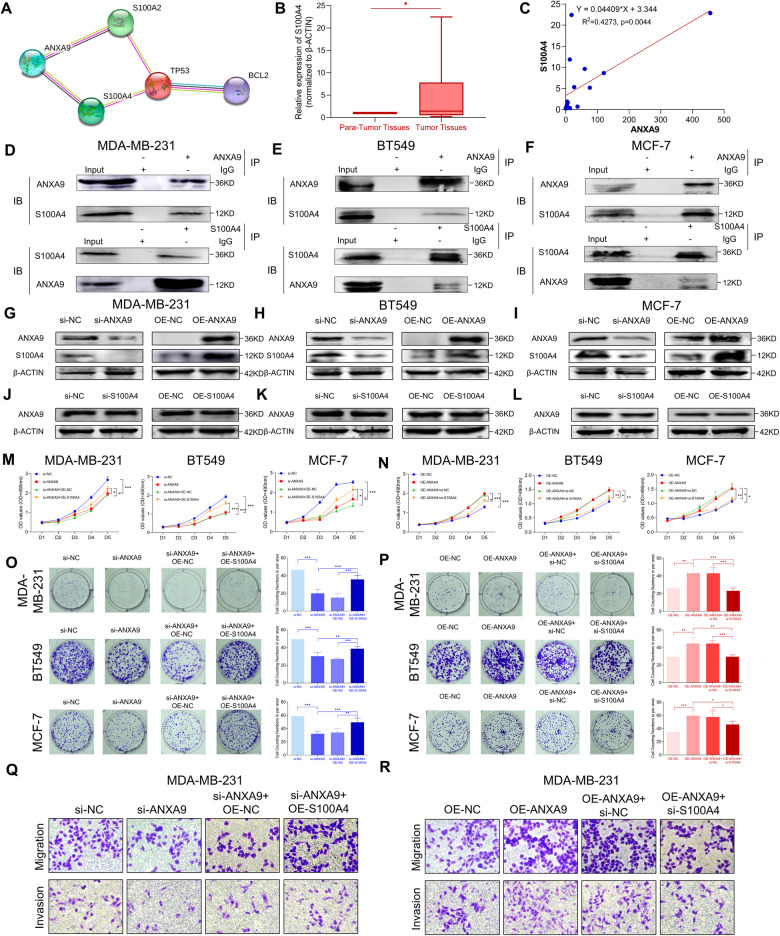
S100A4 is a downstream regulator of ANXA9. **A** The analysis from STRING suggests that ANXA9 interacts with S100A4; **B**, **C** S100A4 is highly expressed in BC and positively correlated with the expression of ANXA9; **D**–**F** co-IP experiments demonstrated the interaction between ANXA9 and S100A4; **G**–**I** ANXA9 affected S100A4 in protein level; **J**–**L** S100A4 have no influence on ANXA9 protein expression; **M**, **N** The rescue experiments revealed the S100A4 could recruit the cellular function which affected by ANXA9 via MTT assays; **O**, **P** The rescue experiments revealed the S100A4 could recruit the cellular function which affected by ANXA9 via colony formation assays. **Q**, **R** The rescue experiments revealed the S100A4 could recruit the cellular motility function which is affected by ANXA9 via migration and invasion assays. *p < 0.05, **p < 0.01, ***p < *0.001*. All of the experiments were replicated for three times.

To elucidate the potential role of ANXA9 in BC, we used the STRING (https://cn.string-db.org/) database to predict potential interacting proteins with ANXA9. As Fig. 4A depicts, ANXA9 could interact with S100A4 in BC. We found that S100A4 was also highly expressed in BC tissues (Fig. 4B), and there was a positive correlation between the expression of ANXA9 and S100A4 (Fig. 4C). Co-IP experiments in three BC cell lines also showed ANXA9-S100A4 interactions (Fig. 4D–F).

To determine whether ANXA9 could regulate S100A4 expression in BC cells, we manipulated the expression of ANXA9 as shown in Fig. 4G–I. The expression of S100A4 altered with the downregulation or upregulation of ANXA9 in BC cells. However, when we changed the expression of S100A4 in BC cells, there was no impact on ANXA9 expression (Fig. 4J–L), indicating ANXA9 as the upstream regulator of S100A4 in BC. We conduct the MTT assay, colony formation assay and transwell assays to perform rescue experiments. The effect of si-ANXA9 could be reversed by upregulation of S100A4 through overexpression of S100A4 (Fig. 4M, O and Q). The effect of OE-ANXA9 could be blocked by downregulation of S100A4 (Fig. 4N, P and R). Our data revealed that ANXA9 could interact with S100A4 in BC.

### ANXA9 regulates the apoptosis in breast cancer via p53 pathway

**Fig. 5:**
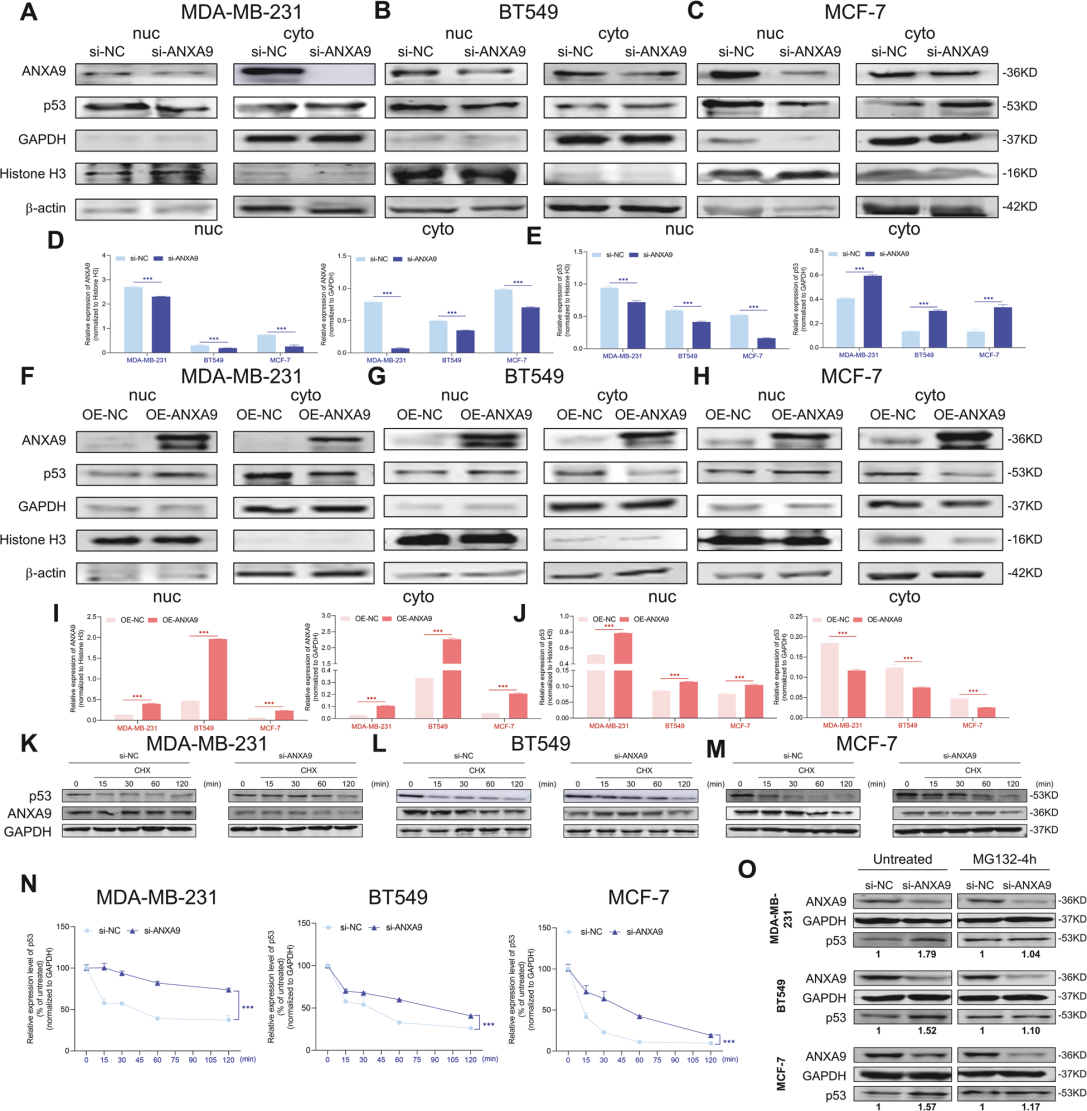
ANXA9 regulates the apoptosis in breast cancer via p53 pathway. **A**–**E** Si-ANXA9 decreased p53 expression in nucleus and increased it in cytoplasm; **F**–**J** OE-ANXA9 increased p53 expression in nucleus and decreased it in cytoplasm; **K**–**O** Si-ANXA9 treatment weakened the time-dependent ubiquitin-proteasome degradation of p53 in BC cells in response to CHX and MG-132 treatment (relative gray values). ***p < *0.001*. All of the experiments were replicated for three times.

To further understand how the ANXA9 regulates p53 expression in BC cells, we divided and collected the proteins from nuclear and cytoplasm sections for confirmation. Si-ANXA9 decreased p53 expression in nucleus and promoted p53 in nucleus transfer into cytoplasm (Fig. 5A–E). ANXA9 overexpression increased nuclear p53 expression and decreased cytoplasmic p53 expression (Fig. 5F–J). After treatment of CHX, the half-life of p53 was increased after ANXA9 downregulation and had a time-related trend (Fig. 5K–N). Furthermore, we used the MG132 to inhibit proteasome and found that p53 protein level in the siRNA group was decreased compared to the control group (Fig. 5O). The results show that decreasing ANXA9 blocks the degradation of p53 via ubiquitin-proteasome pathway and caused p53 to become retentive in the cytoplasm.

### ANXA9 regulates breast cancer progression through AKT/mTOR/STAT3 pathway

**Fig. 6:**
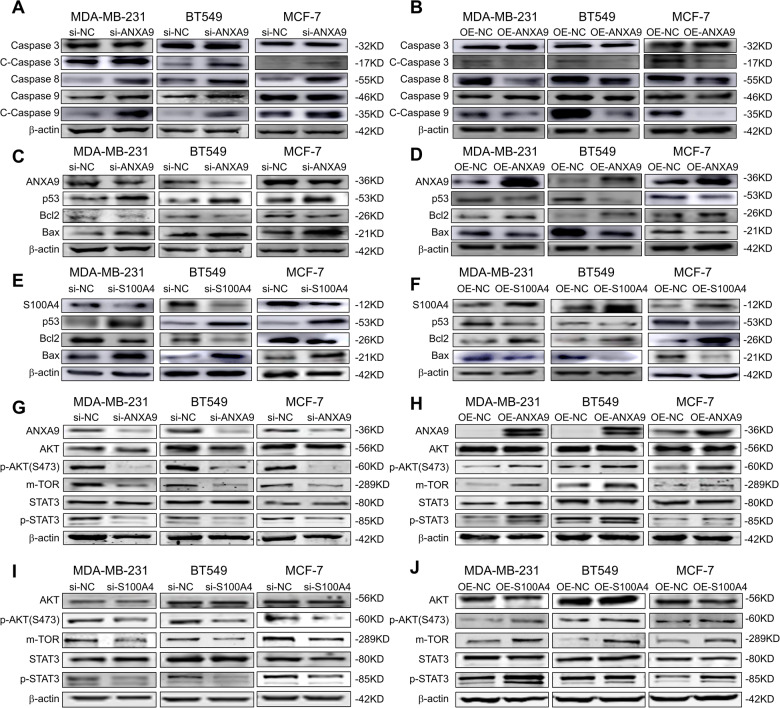
ANXA9 and S100A4 regulates apoptosis through modulating STAT3. **A**, **B** ANXA9 regulated protein expression of cleaved-caspase3 and cleaved-caspase9 in BC cells; **C**, **D** ANXA9 regulated protein expression of p53, Bcl-2 and Bax in BC cells; **E**, **F** S100A4 affected the protein expression of p53, Bcl-2 and Bax in BC cells; **G**, **H** ANXA9 affected the AKT/STAT3/ mTOR signal pathway; **I**, **J** S100A4 affected the AKT/STAT3/ mTOR signal pathway. All of the experiments were replicated for three times.

Western blot results revealed that cleaved-caspase 3, caspase 8, and cleaved-caspase 9 expression was increased by downregulation of ANXA9 and decreased by overexpression of ANXA9 respectively, in three BC cell lines (Fig. 6A, B). Besides, si-ANXA9 increased p53 protein expression, decreased Bcl-2 protein expression, and increased Bax protein expression (Fig. 6C). Overexpression of ANXA9 decreased p53 protein expression, increased Bcl-2 protein expression, and decreased Bax protein expression (Fig. 6D). Changing the expression of S100A4 in BC has the same regulation effect with ANXA9 (Fig. 6E, F). Our results revealed that ANXA9 could interact with S100A4 to regulate the p53/Bcl-2 apoptotic signal pathway.

However, research on regulating p53/Bcl-2 by ANXA9/S100A4 is limited. We analyzed the potential signal pathway through the website (https://www.cellsignal.cn/pathways). We found that ANXA9/S100A4 could regulate AKT/mTOR/STAT3, affecting the p53 signal pathway. In three BC cell lines, downregulation of ANXA9 protein levels decreased phosphorylation of AKT, mTOR expression, and STAT3 phosphorylation, whereas the total protein level of AKT and STAT3 was not affected (Fig. 6G). ANXA9 overexpression increased phosphorylation of AKT, mTOR expression, and STAT3 phosphorylation in three BC cell lines but did not affect total protein levels of AKT and STAT3 (Fig. 6H). The S100A4 could either regulate the phosphorylation of AKT, mTOR expression, or STAT3 phosphorylation (Fig. 6I, J).

### ANXA9 transfers S100A4 out of breast cancer cells via a phosphorylation state

**Fig. 7:**
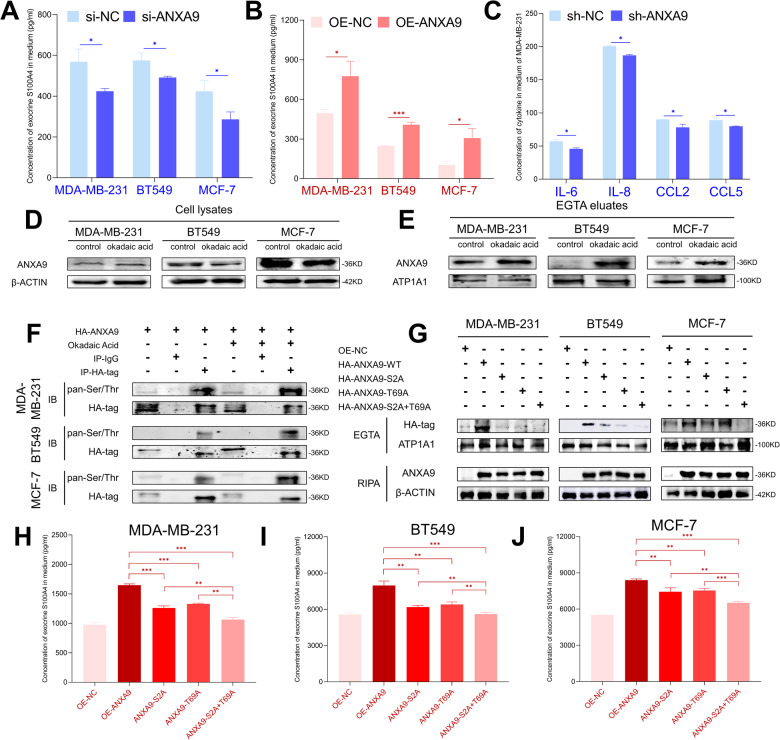
Self-phosphorylation of ANXA9 assists S100A4 in transferring from intracellular to extracellular. **A**, **B** S100A4 content in extracellular medium changed after that cells treated with alteration of ANXA9; **C** Knocking down ANXA9 in MDA-MB-231 cells resulted in reducing secretion of IL-6, IL-8, CCL2, and CCL5 in extracellular medium; **D**, **E** Okadaic acid had no influence on the protein expression of ANXA9 in cytoplasm of BC cells but affected ANXA9 expression on the cell membrane; **F** Co-IP showed that over-expressing ANXA9 and okadaic acid treatment increased ANXA9 phosphorylation on the cell membrane; **G** Three groups of ANXA9 mutant plasmid: S2^mutant^, T69^mutant^ and S2/T69^mutant^ decreased ANXA9 expression on the cell membrane compared with transfecting wild type ANXA9 plasmids; **H**–**J** ELISA results showed that S100A4 content decreased in extracellular medium of the S2^mutant^ or T69^mutant^ groups and decreased more in S2/T69^mutant^ group compared with wild type ANXA9 plasmids. *p < 0.05, **p < 0.01, ***p < *0.001*. All of the experiments were replicated for three times.

It has been reported that S100A4 could be exocrine and some cytokines, such as IL-6, IL-8, CCL2, and CCL5 can be recruited by exocrine S100A4 to control metastasis via angiogenesis [15]. The results showed that when ANXA9 changed, the exocrine S100A4 content in the cellular medium also changed, consistently (Fig. 7A, B). In addition, the levels of IL-6, IL-8, CCL2, and CCL5 were reduced after treatment with sh-ANXA9 (Fig. 7C).

To investigate why ANXA9 transfers S100A4 out of BC cells, we treated BC cells with okadaic acid, a phosphatase inhibitor, to increase the level of phosphorylation of ANXA9. Based on our results, ANXA9 expression in cytoplasm changed minimally (Fig. 7D), but increased on the membrane of cells (treated with EGTA solution) (Fig. 7E). Then, we overexpressed ANXA9 in BC cells, and treated them with okadaic acid. Finally, we found that the phosphorylation level of ANXA9 increased, indicating that ANXA9 is phosphorylated on the membrane (Fig. 7F). We screened the database of PhosphoSitePlus (https://www.phosphosite.org/homeAction.action) to explore the potential phosphorylation sites of ANXA9 and found that could exist at the second serine (abbreviated as S2) or at the 69th threonine (abbreviated as T69). To confirm that, we constructed site-mutant plasmids of ANXA9 on the S2 site (S2 ^mutant^), T69 site (T69 ^mutant^) and both (S2/T69 ^mutant^). The phosphorylation level of ANXA9 decreased in either the S2 ^mutant^ or T69 ^mutant^ group and almost disappeared in the S2/T69 ^mutant^ (Fig. 7G) when compared to the wild type. Furthermore, the exocrine S100A4 decreased in either the S2 ^mutant^ or T69 ^mutant^ group and decreased significantly in the S2/T69 ^mutant^ group (Fig. 7H–J). The results confirmed that the phosphorylation level of ANXA9 on the membrane was the reason for the phosphorylation of S2/T69 sites and that phosphorylation of ANXA9 could transport S100A4 out of BC cells.

### ANXA9 mediates S100A4 to regulate breast cancer metastasis

**Fig. 8:**
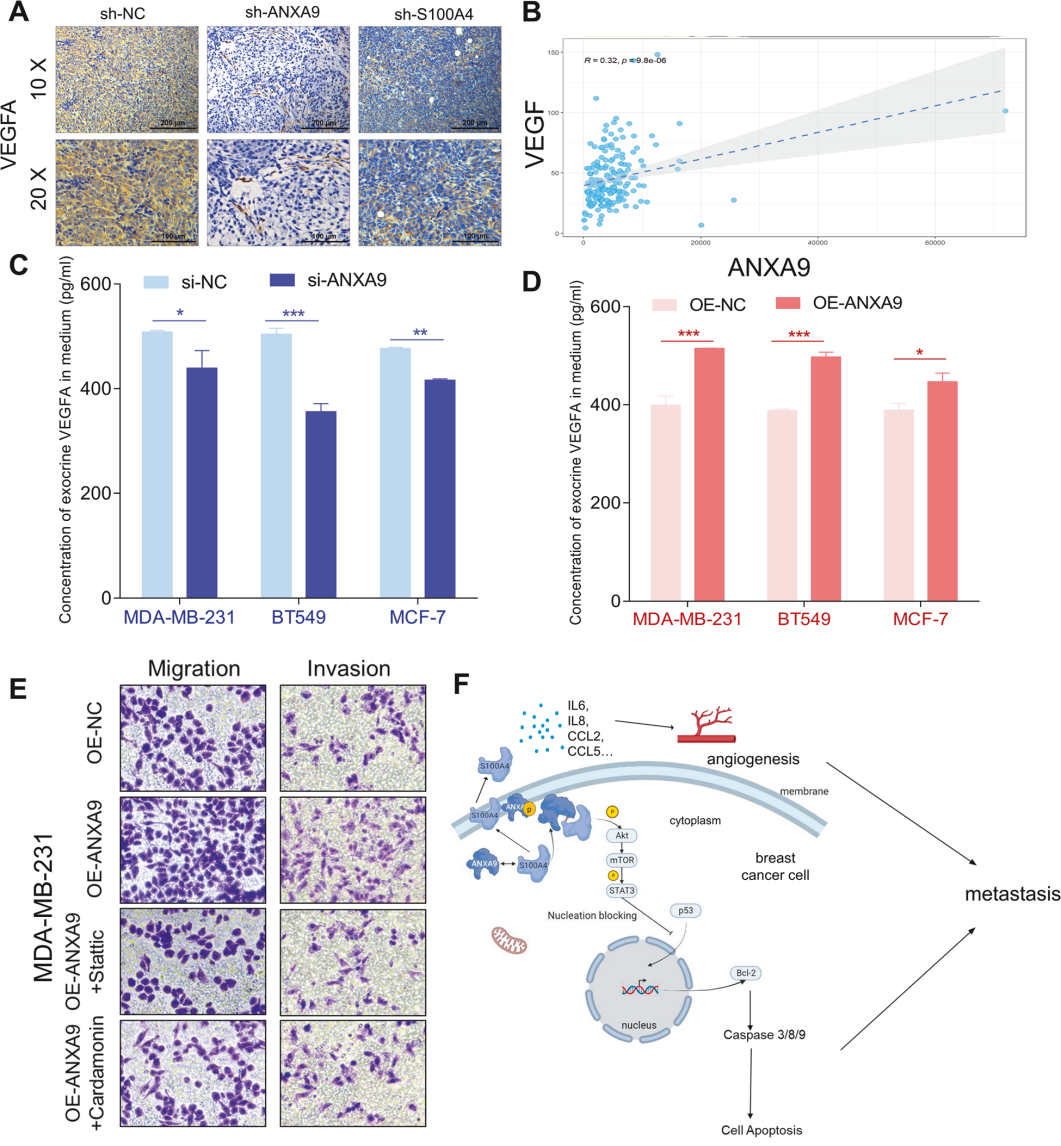
ANXA9 enhances VEGF expression and promotes breast cancer metastasis. **A** Tumors from mice xenografts stained with IHC staining showed a decrease in VEGF expression in sh-ANXA9 and sh-S100A4 groups; **B** Analysis of bioinformatics data from TCGA revealed that ANXA9 expression in MBC is positively correlated with VEGF expression (R = 0.32, *p* = 9.8e-06); **C** Down-regulated ANXA9 in BC cells resulted in decreasing secretion of VEGF in extracellular medium; **D** Up-regulated ANXA9 in BC cells resulted in increasing secretion of VEGF in extracellular medium; **E** The rescue experiments revealed the inhibitors of STAT3 (Stattic and Cardamonin) could recruit the cellular motility function which affected by ANXA9 via migration and invasion assays; **F** The sketch map of the regulation and mechanism of ANXA9 mediated BC metastasis. *p < 0.05, **p < 0.01, ***p < *0.001*. All of the experiments were replicated for three times.

The expression of VEGFA in the xenograft tumor also revealed that sh-ANXA9 and sh-S100A4 could decrease it (Fig. 8A). We evaluated the correlation between ANXA9 and VEGFA and found a positive correlation in BC (Fig. 8B). We also checked the concentration of VEGFA in the medium of MDA-MB-231 cells. Our results showed that downregulation of ANXA9 decreased the concentration of VEGFA and upregulation of ANXA9 increased the concentration of VEGFA (Fig. 8C, D). In addition, we performed the rescue experiments of treating BC cells with Stattic (STAT3 inhibitor) and Cardamonin (AKT/mTOR/STAT3 signal pathway inhibitor), the effect of overexpression of ANXA9 could be rescued by these two inhibitors (Fig. 8E). Our results suggest that, on one side, ANXA9 could regulate AKT/mTOR/STAT3 to regulate p53 signal pathway, participating BC metastasis and on the other side, ANXA9 facilitates the transfer of S100A4 out of BC cells, recruiting several cytokines to help cancer metastasis via angiogenesis (Fig. 8F).

## Discussion

ANXA9 belongs to the annexin (ANX) family, and Annexin proteins are structurally characterized by their core domains, which consist of a series of four repeated annexin sequences, each containing a calcium-binding motif that mediates reversible calcium-dependent binding with negatively charged phospholipids [16], such as phosphatidylserine, phosphatidylglycerol, and phosphatidylinositol [17]. Functionally, annexin, as a membrane binding protein regulated by calcium [18, 19], not only participates in cell membrane formation [20], regulates transmembrane transport [21] and calcium ion aggregation inside and outside the membrane [22], but also regulates physiological functions such as cell cycle and apoptosis [23, 24]. There are few studies reports on ANXA9, with most studies focusing on digestive system tumors [4, 25]. The research landscape concerning ANXA9 in the context of BC remains relatively unexplored. However, a solitary study has suggested a potential association between ANXA9 and BC bone metastasis, as inferred from data analysis [26].

In our functional experiments, knockdown of ANXA9 in vivo and in vitro could inhibit the progression of BC. Considering that the ANX family could regulate apoptosis [24], we conducted a series of experiments to examine whether ANXA9 could regulate BC apoptosis. Our study indicated that downregulation of ANXA9 could increase BC cellular early apoptosis rate (Fig. 3I, J). Western blot results revealed that cleaved-caspase3, caspase8, and cleaved-caspase9 expression was increased by downregulation of ANXA9 and decreased by overexpression of ANXA9 respectively in MDA-MB-231, BT549, and MCF-7 cells (Fig. 6A, B). To figure out the potential mechanism of ANXA9 regulating apoptosis of BC, we found p53 could function as an essential key regulator [27]. The expression of p53 and Bcl-2/Bax proteins of BC could be regulated by ANXA9, as shown in Fig. 6C, D. Furthermore, ANXA9 mediated p53 transportation from nuclear to the cytoplasm. CHX was used to block protein synthesis to determine whether the increased p53 level was caused by the downregulation of ANXA9 [28]. The half-life of p53 increased following the downregulation of ANXA9 after CHX treatment, and this trend was time-related. Furthermore, MG132 was used to inhibit proteasomes, and found that the p53 protein level in the siRNA group was decreased compared to the control group. Based on these studies, ANXA9 decreases could block p53 degradation by the proteasome pathway and cause the protein to remain in the cytoplasm.

To further elucidate the possible mechanism of ANXA9 in BC, we analyzed the STRING (https://cn.string-db.org/) database for potential interacting proteins. As we found in the database, S100A4 could be a target protein of ANXA9. Interestingly, S100A4 was highly expressed in BC and correlated positively with ANXA9. Co-IP experiments in three BC cells also demonstrated ANXA9 and S100A4 interacted (Fig. 4D–F). Furthermore, ANXA9 could regulate the expression of S100A4, but instead, the S100A4 could not regulate the ANXA9’s expression (Fig. 4G–L). Our results demonstrated that ANXA9 was the upstream regulator of S100A4 in BC. The rescue experiments confirmed that S100A4 reversed the effects of ANXA9 (Fig. 4M–R). Hence, S100A4 acted as an important regulator of ANXA9’s ability to regulate BC progression. Consequently, the biological functions of S100A4 in BC have also been explored and presented in Supplement Fig. 1. We found that ANXA9 mediated S100A4 regulating AKT/mTOR/STAT3, affecting the p53/Bcl-2 signal pathway. In numerous ways, signal converters and activators of transcription (STAT3) participate in BC occurrence and development. Based on studies, STAT3 is constitutively activated in all BC subtypes, and has the highest correlation with TNBC [29]. As a result of activation of STAT3, caspases are inhibited, and cell apoptosis is blocked through the regulation of downstream targets like survivin, Bcl-2, Bcl-xL, and Mcl-1 [30]. Moreover, STAT3 is implicated in neovascularization in TNBC, promoting angiogenesis in TNBC by upregulating vascular endothelial growth factor and other downstream target genes [31].

In the S100 protein family, S100A4 participates in multiple phenotypic changes that affect tumor cells, including promoting the proliferation of acute leukemia cells [32], increasing the metastatic ability of ovarian cancer cells [33], and promoting the degradation and remodeling of extracellular matrix in the microenvironment of breast tumors [34]. Moreover, S100A4 has intracellular binding functions of binding to tumor suppressor p53, disrupting its stability, and inhibiting cell apoptosis [35]; It also has extracellular functions of secreting into the extracellular matrix, acting on membrane receptors, activating the non-receptor type tyrosine protein kinase 2 (JAK2) [36]. In addition, S100A4 can affect BC prognosis by changing BC-related fibroblastic cell habits [37]; A high expression of S100A4 in BC has also been linked to axillary lymph node metastasis [38].

Additionally, we found that ANXA9 could transfer the S100A4 out of cancer cells (Fig. 7A, B) and down regulation of ANXA9 could decreased the cytokines of IL-6, IL-8, CCL2, and CCL5 (Fig. 7C). To investigate the mechanism behind ANXA9’s ability to transfer the S100A4 out of cancer cells, we treated BC cells with okadaic acid (a phosphatase inhibitor) to increase ANXA9 phosphorylation. Based on our results, ANXA9 expression in the cytoplasm has changed minimally (Fig. 7D). However, its expression increased on the membrane of cells when the cells were treated with the EGTA method to collect the protein of cell membrane (Fig. 7E). It is well known that proteins are macromolecular substances and that exocytosis is the main method of releasing proteins, which is neither active nor passive [39]. ANXA2 has been reported to translocate from the cytoplasm to the cell membrane and flip from inside to outside during temperature stress, relying on S100A10 and its own tyrosine phosphorylation, forming a protein secretion pathway different from the classical exocytosis form and affecting the fibrinolytic system in organisms [40].

To verify whether S100A4 could be transferred out of BC cells based on the phosphorylation level of ANXA9, we constructed phosphorylation site-mutant plasmids. The results showed that the phosphorylation level of ANXA9 decreased in either the S2 ^mutant^ or T69 ^mutant^ group and almost disappeared in S2/T69 ^mutant^ (Fig. 7G) compared to the wild type of ANXA9 group. The exocrine S100A4 also decreased in either the S2 ^mutant^ or T69 ^mutant^ group, and significantly decreased in S2/T69 ^mutant^ group (Fig. 7H-J). The results confirmed that the phosphorylation level of ANXA9 on the membrane was the reason for the phosphorylation of S2/T69 sites and that the phosphorylation of ANXA9 could transfer S100A4 out of BC cells.

According to studies, BC cells express greater amounts of IL-6, which functions as a cancer promoter [41]. The secreted IL-6 and IL-8 could promote angiogenesis and favor the growth and spread of the disease [42]. In addition, CCL2 and CCL5 were also reported to promote angiogenesis and mediate the breast metastatic processes [43]. VEGF signaling was known as a famous promoter of the MBC and [44] had a significantly positive correlation with high vascular counts and positive axillary lymph nodes [45]. A strong association exists between serum IL-6, VEGF, and BC development. These markers can be used to diagnose BC at an early stage [46]. The expression of VEGFA in the xenograft tumor also revealed that sh-ANXA9 and sh-S100A4 could decrease the expression of VEGFA (Fig. 8A). There was a positive correlation between ANXA9 and VEGFA in BC (Fig. 8B). ANXA9 could also regulate the VEGFA concentration out of BC cells (Fig. 8C, D). Therefore, the ANXA9 could transfer S100A4 out of BC to affect IL-6, IL-8, CCL2, and CCL5 cytokines to mediate BC angiogenesis.

In summary, the results of our study revealed two different mechanisms through which ANXA9 regulates BC progression. On the one hand, ANXA9 regulates AKT/mTOR/STAT3/p53 axis via S100A4 to mediate apoptosis in BC. On the other hand, ANXA9 could transfer S100A4 out of BC cells to mediate angiogenesis to participate in metastasis in BC (Fig. 8C). We anticipate that ANXA9/S100A4 will become a potentially promising therapeutic target for BC interception in the future.

### Supplementary information

Supplement Table 1

Revised supplement materials

Supplement information

Original Data File

## Data Availability

We searched the ONCOMINE database with the term “metastatic breast cancer” and downloaded a series of differentially expressed genes as listed in Supplement Table 1. The clinical parameters characteristics of breast cancer and the expression matrix of ANXA9 in breast cancer were downloaded from TCGA and analyzed with R v4.3.1 software.
