# Supplementary material for: ANXA9 facilitates S100A4 and promotes breast cancer progression through modulating STAT3 pathway
Source: Cell Death Dis. 2024 Apr 12;15(4):260. doi: 10.1038/s41419-024-06643-4 (PMC11014919; doi:10.1038/s41419-024-06643-4)
Supplement: Supplementary file 2 — Revised supplement materials [file 41419_2024_6643_MOESM2_ESM.pdf]

Supplement figure 1 Biological functions of S100A4 in breast cancer cells.

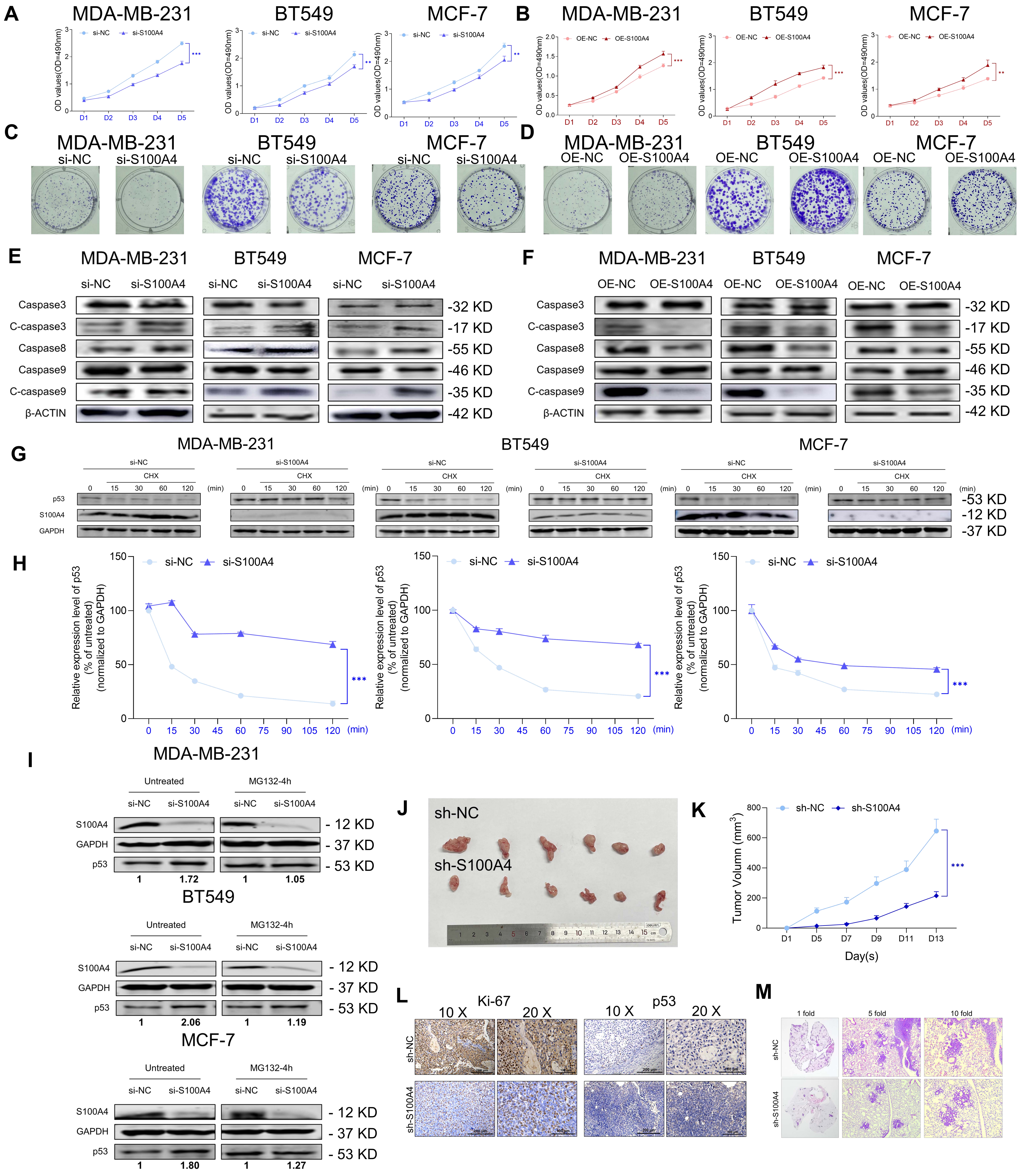

A
MDA-MB-231
BT549
MCF-7
OD values(OD=490nm)
si-NC
si-S100A4
D1
D2
D3
D4
D5
B
OE-NC
OE-S100A4
C
D
E
Caspase3
C-caspase3
Caspase8
Caspase9
C-caspase9
β-ACTIN
-32 KD
-17 KD
-55 KD
-46 KD
-35 KD
-42 KD
F
G
CHX
(min)
0
15
30
60
120
p53
S100A4
GAPDH
-53 KD
-12 KD
-37 KD
H
Relative expression level of p53
(% of untreated)
(normalized to GAPDH)
I
Untreated
MG132-4h
- 12 KD
- 37 KD
- 53 KD
1
1.72
1.05
2.06
1.19
1.80
1.27
J
sh-NC
sh-S100A4
K
Tumor Volumn (mm3)
Day(s)
D7
D9
D11
D13
L
Ki-67
p53
10 X
20 X
M
1 fold
5 fold
10 fold

Supplement data 3. Gray scale analysis for western blots.

Western Blot analysis for Fig. 4G-I

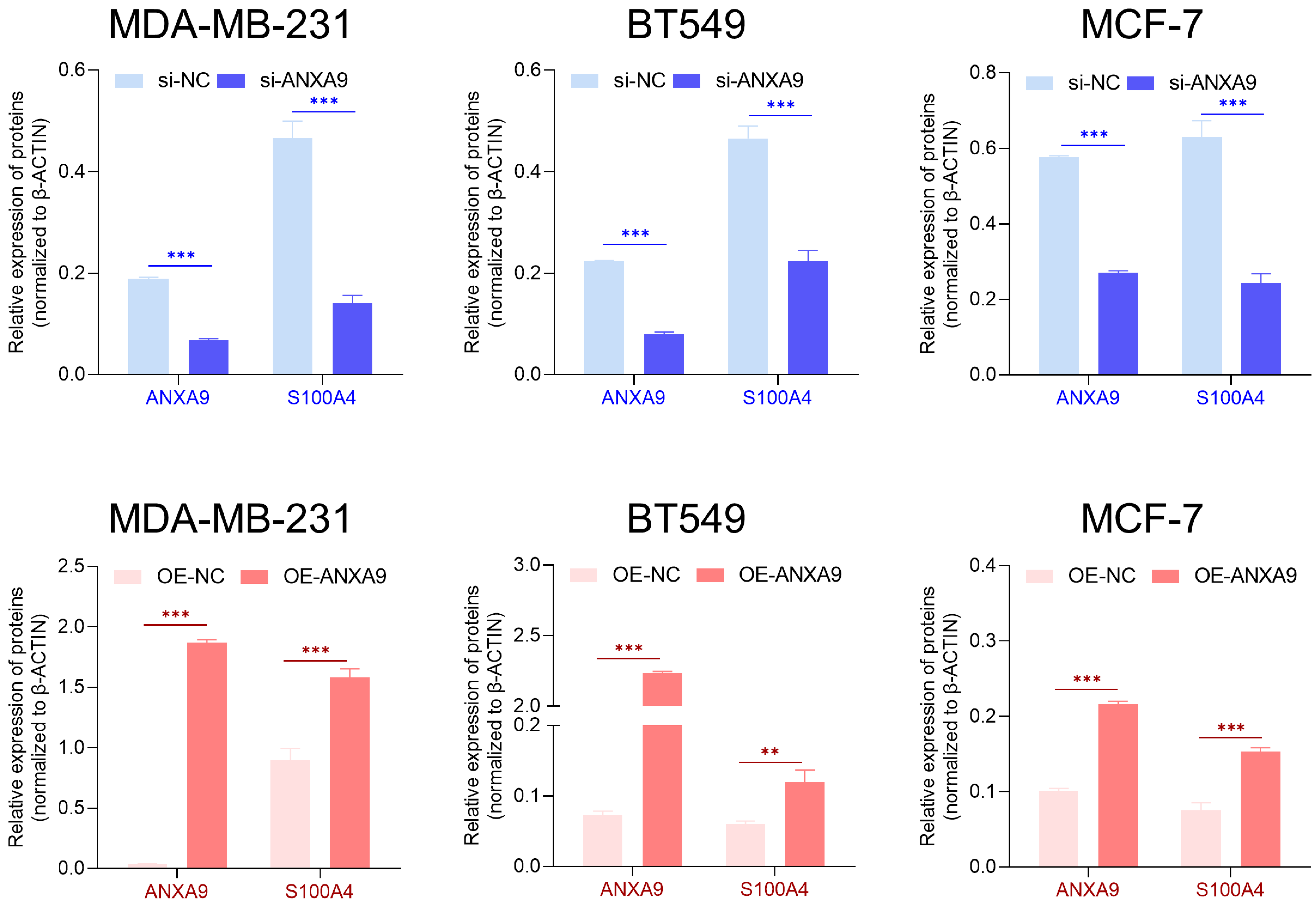

Western Blot analysis for Fig. 4J-L

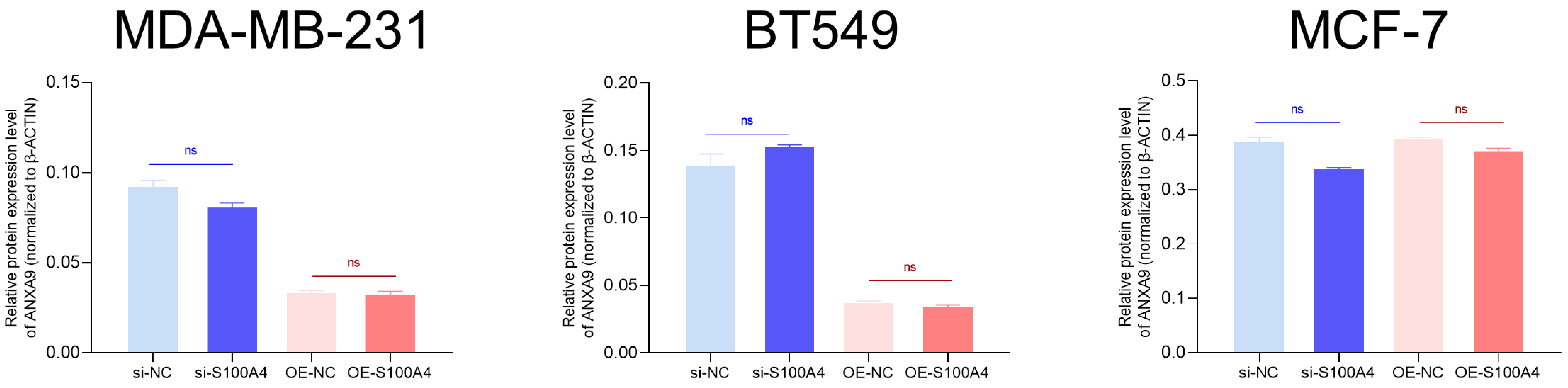

Western Blot analysis for Fig. 5K-M

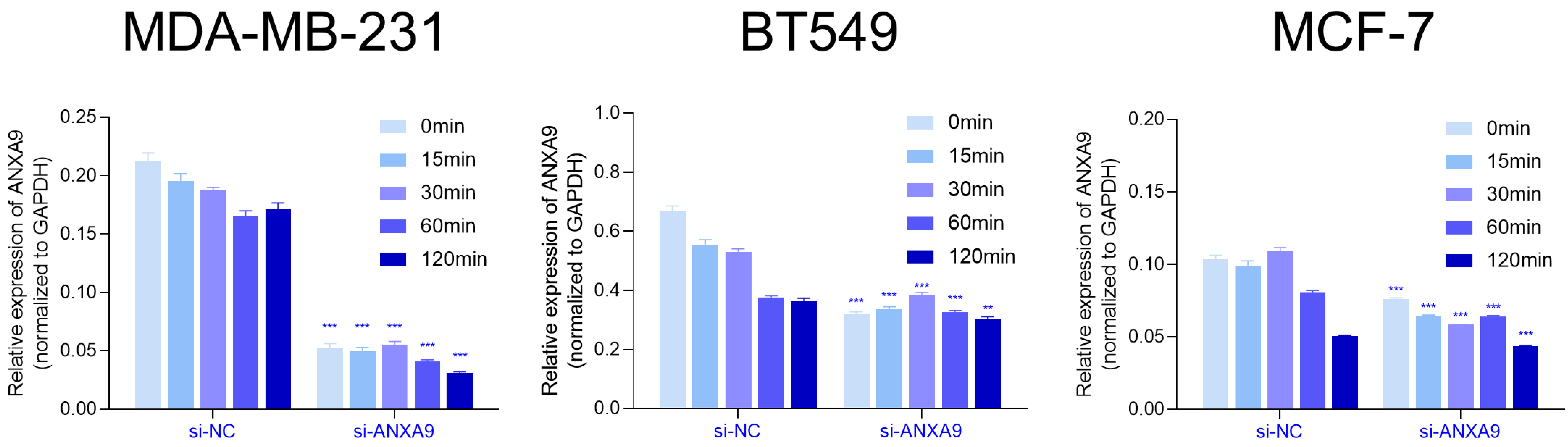

Western Blot analysis for Fig. 6A

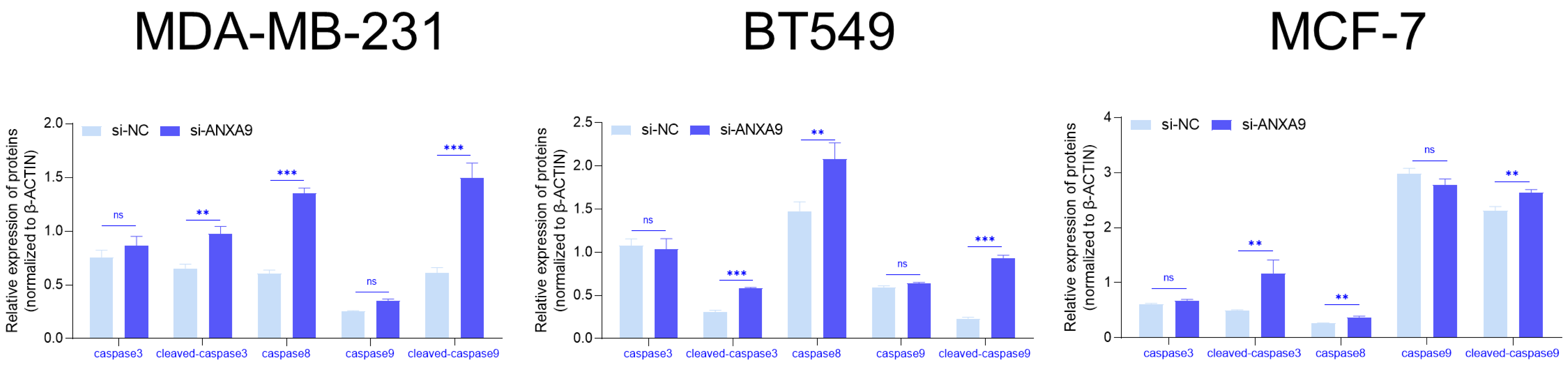

Western Blot analysis for Fig. 6B

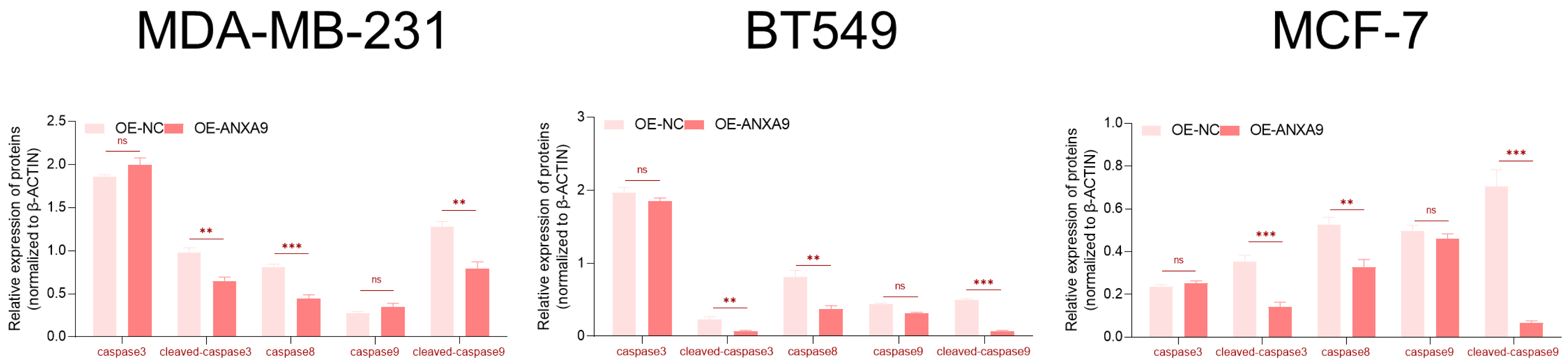

Western Blot analysis for Fig. 6C

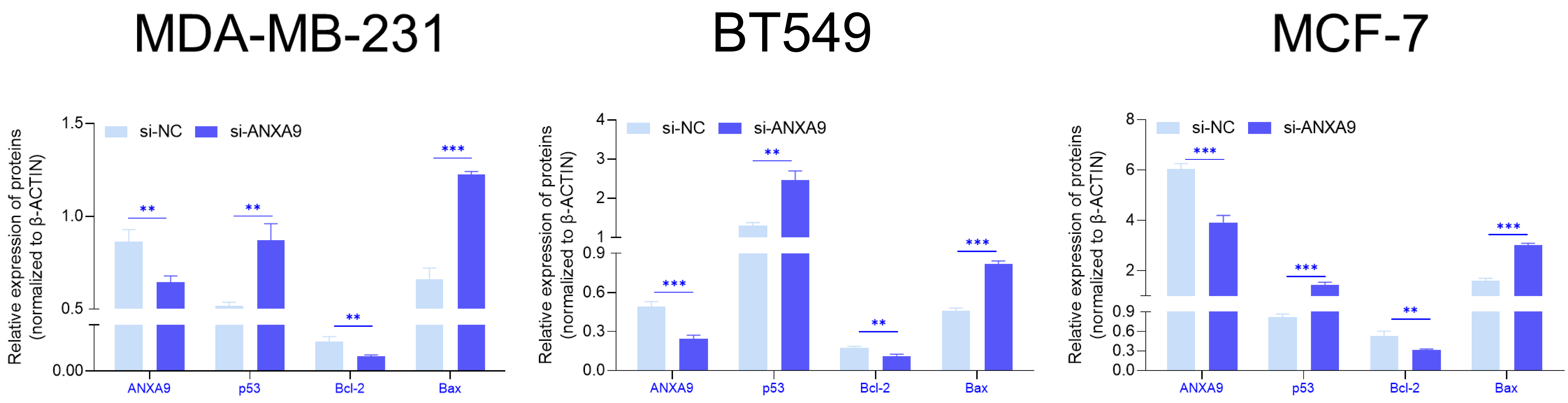

Western Blot analysis for Fig. 6D

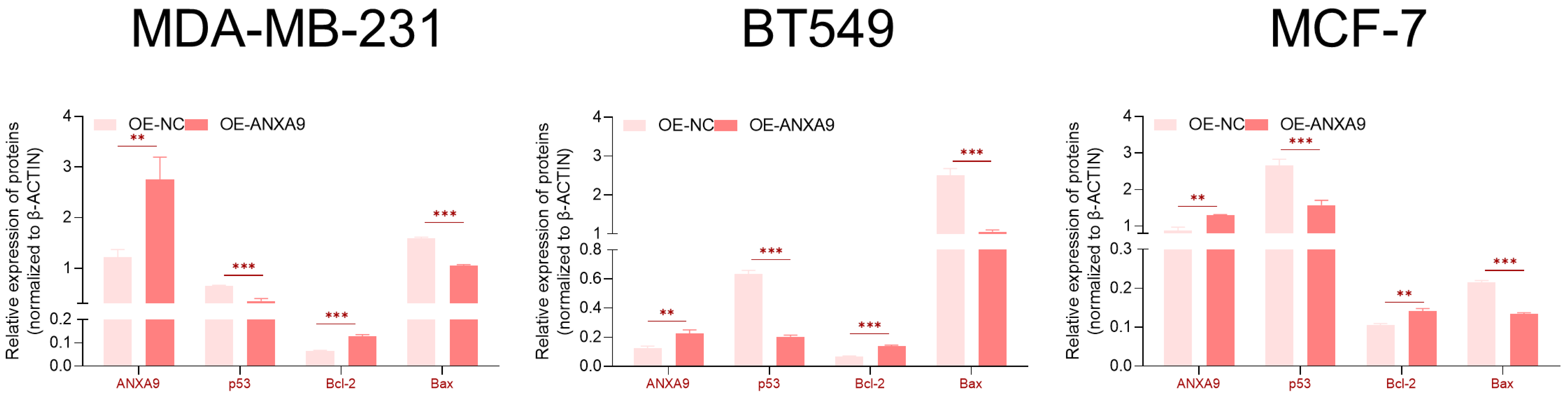

Western Blot analysis for Fig. 6E

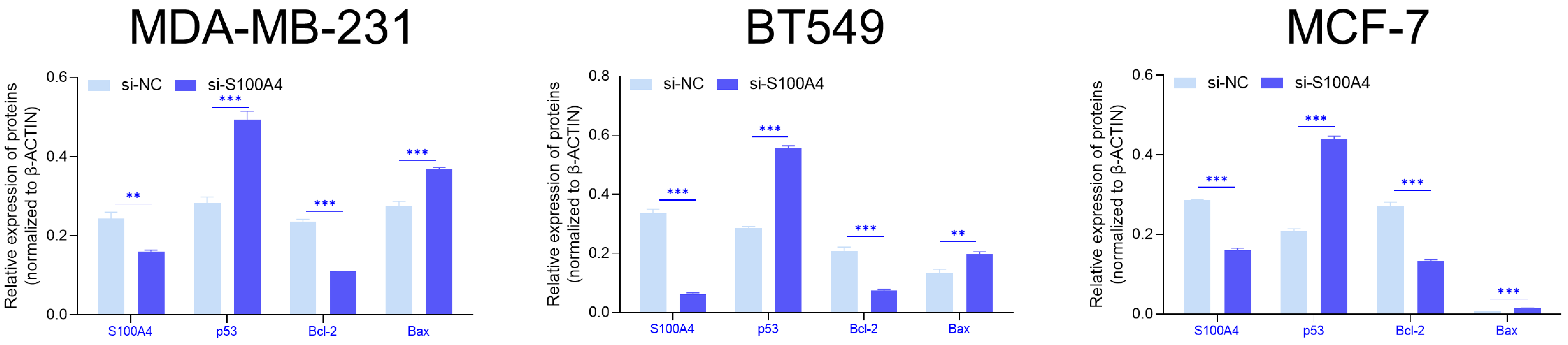

Western Blot analysis for Fig. 6F

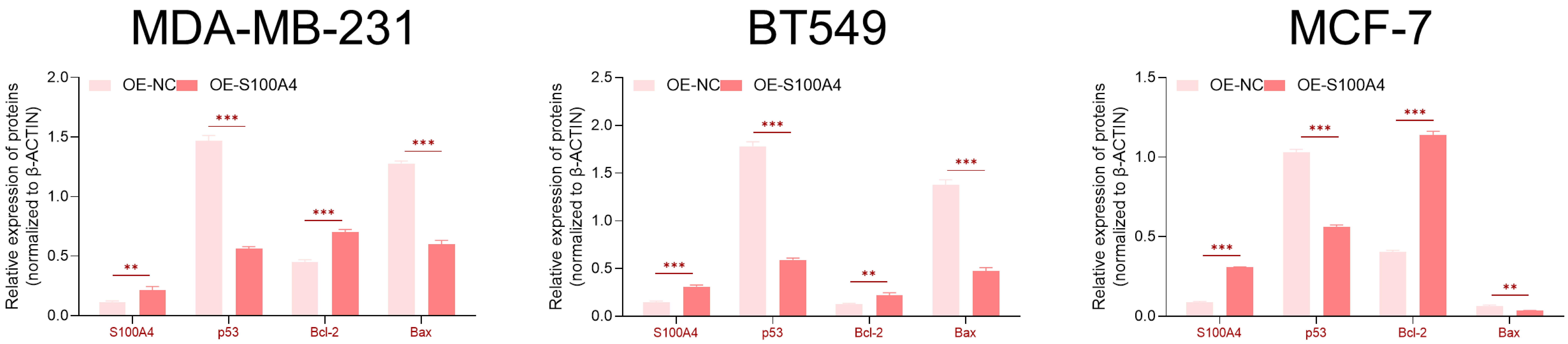

Western Blot analysis for Fig. 6G

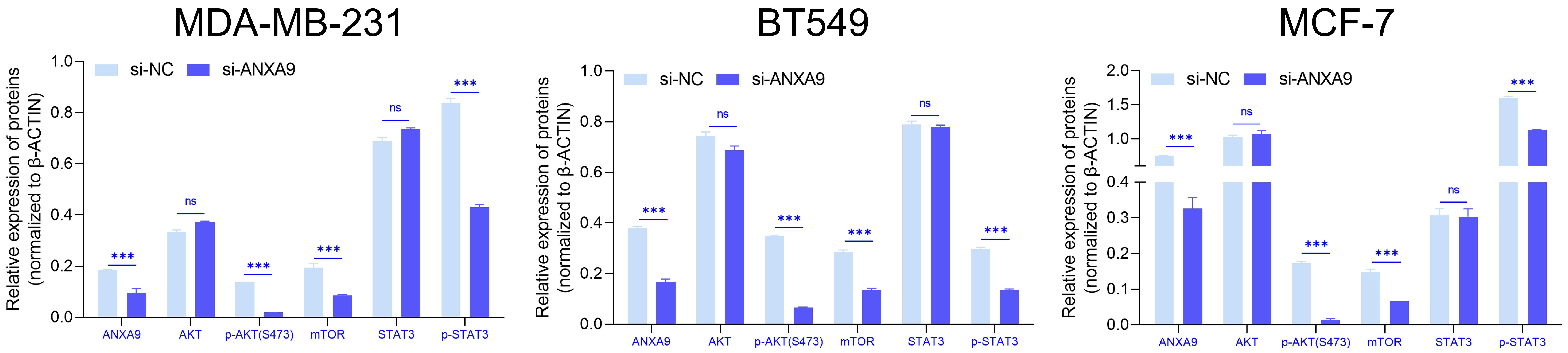

Western Blot analysis for Fig. 6H

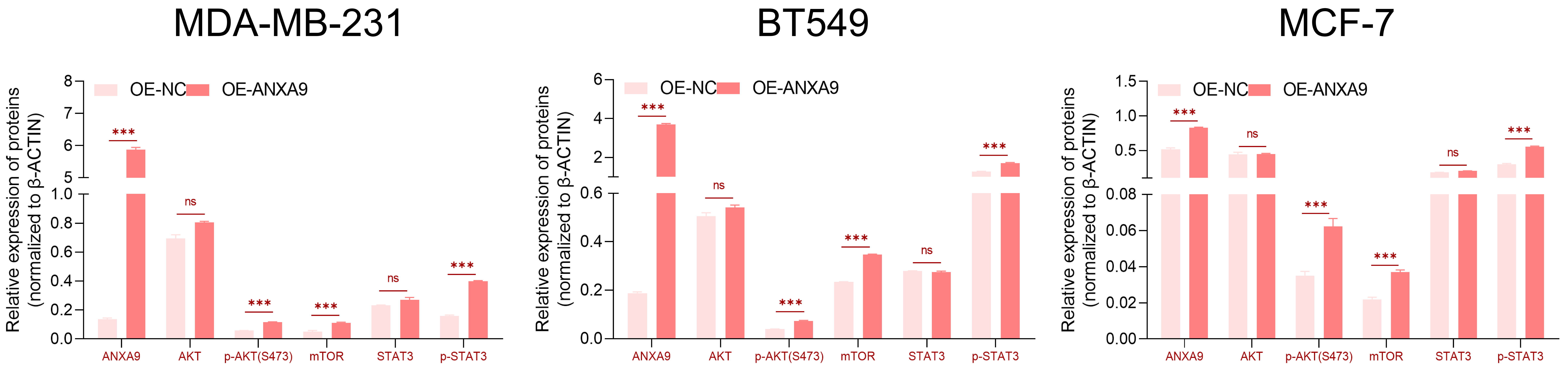

Western Blot analysis for Fig. 6I

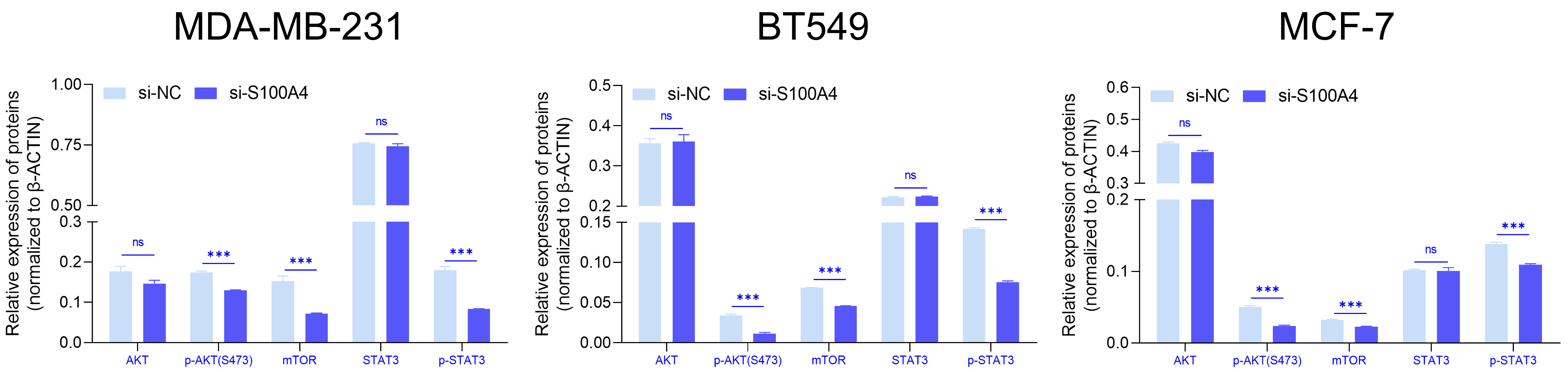

Western Blot analysis for Fig. 6J

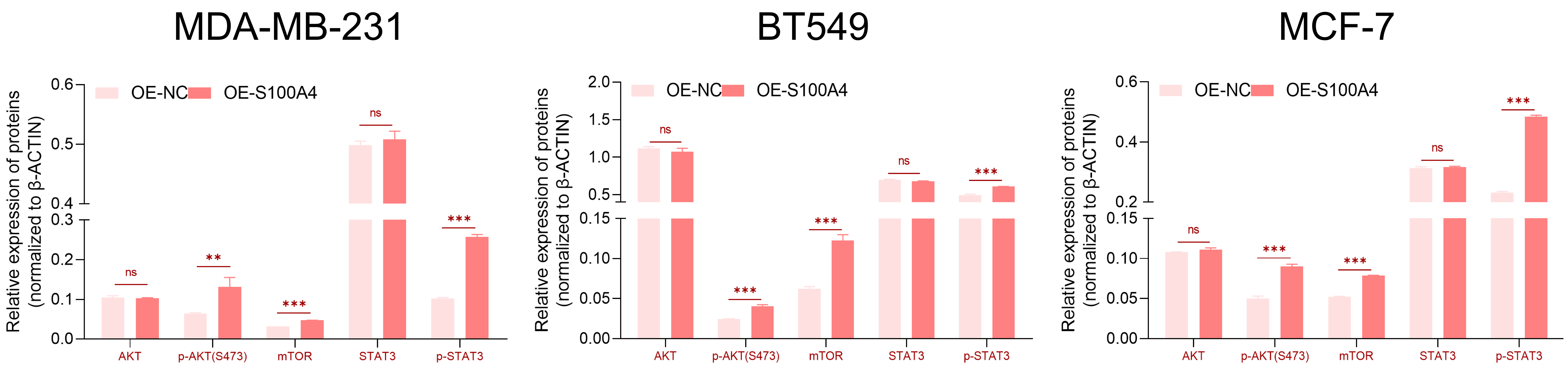

Western Blot analysis for Fig. 7D

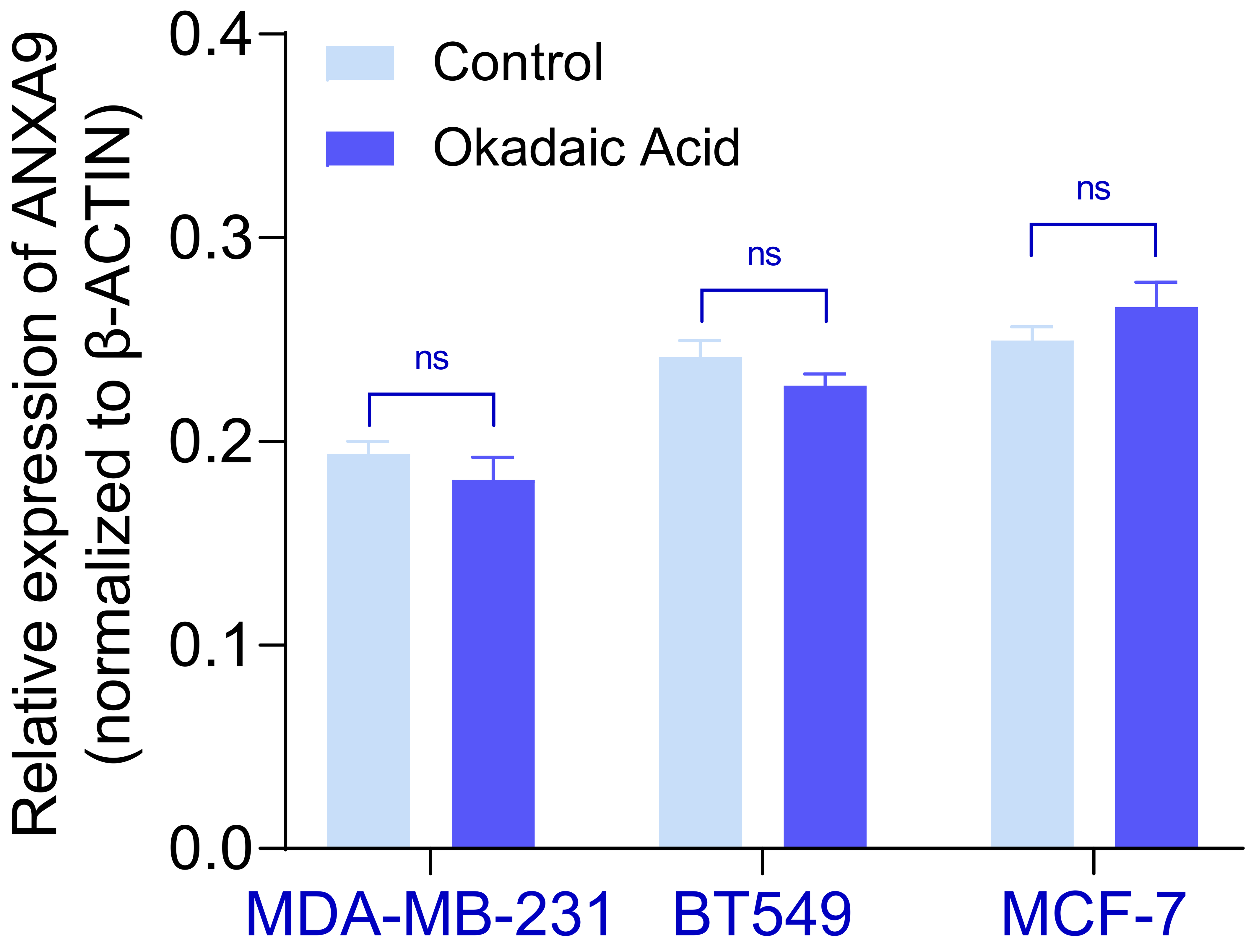

Western Blot analysis for Fig. 7E

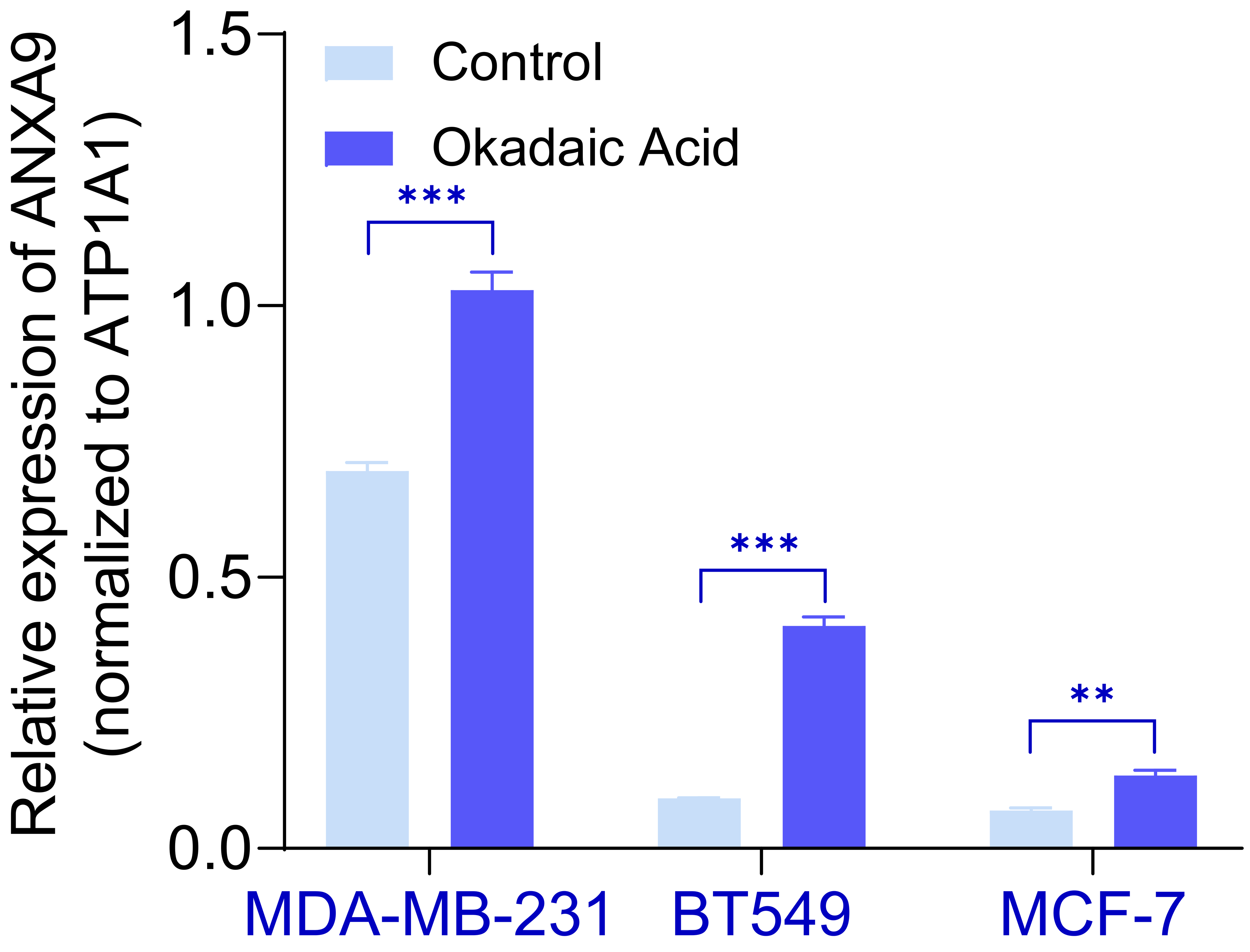

Western Blot analysis for Fig. 7F

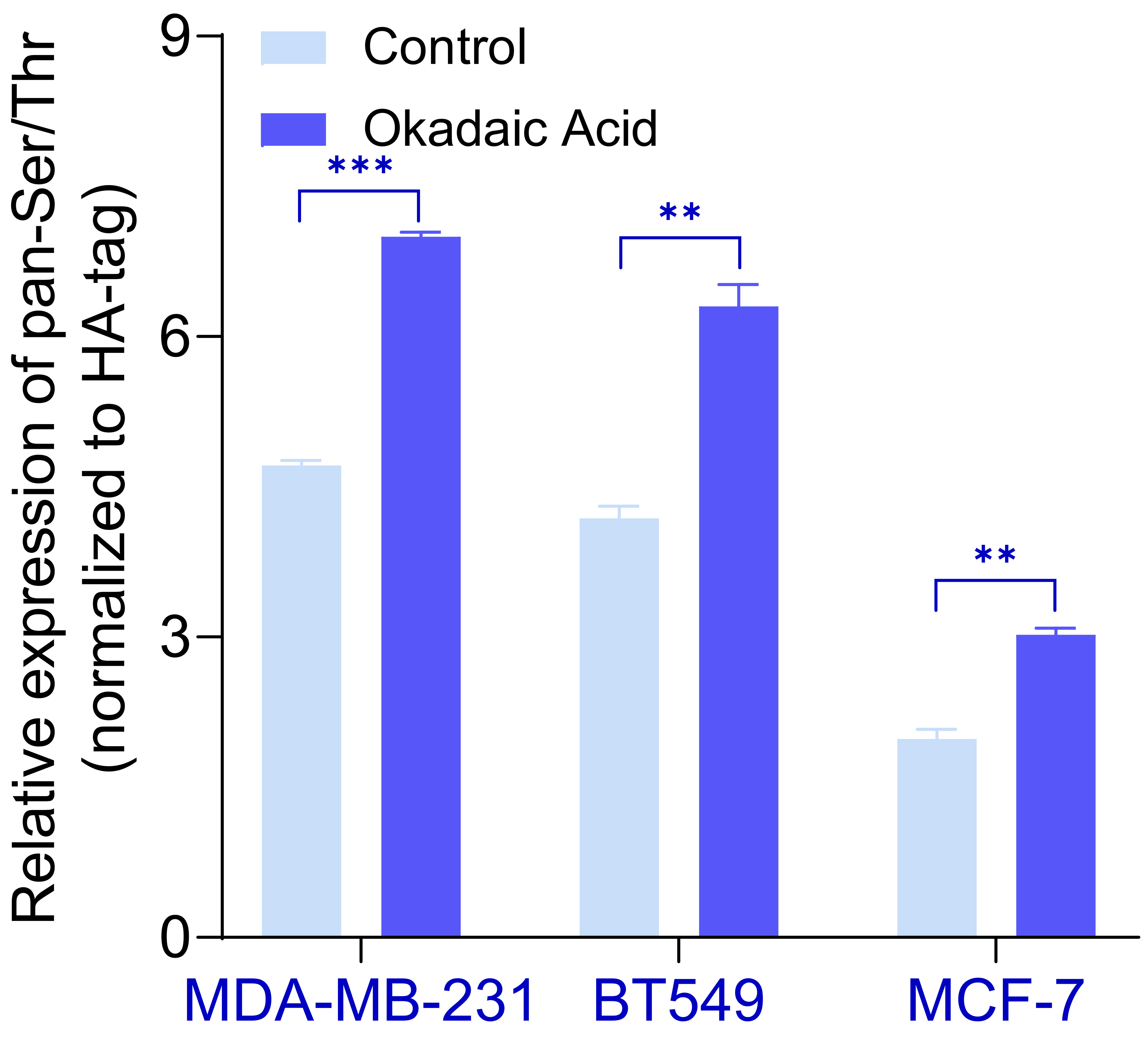

Western Blot analysis for Fig. 7G

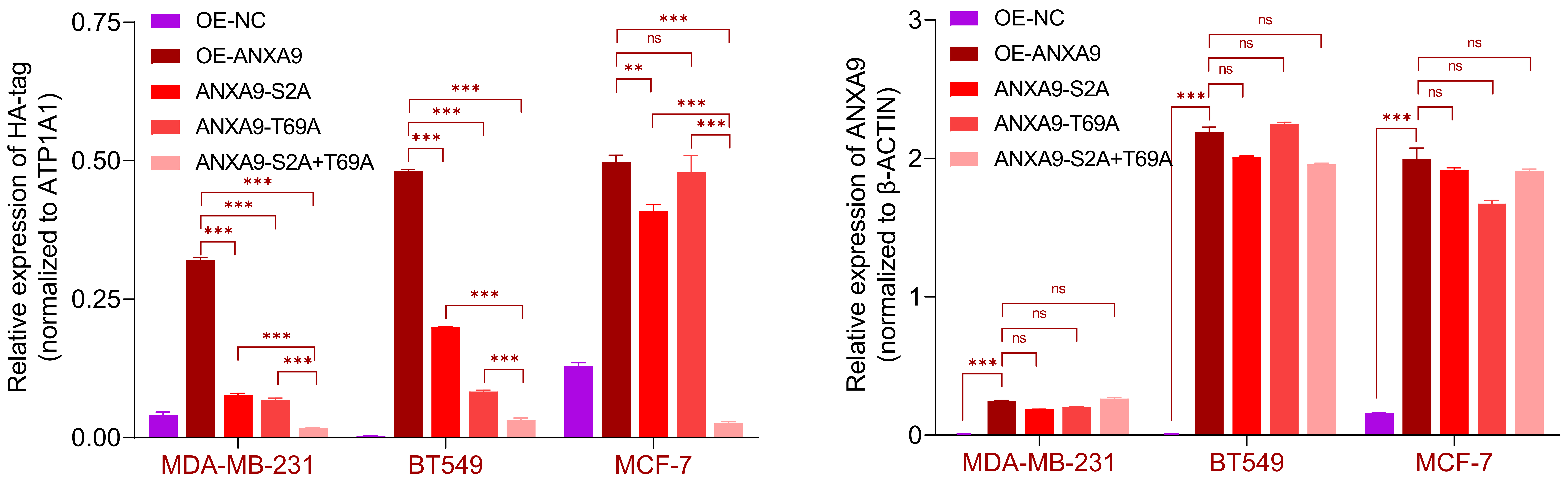

Western Blot analysis for Supplement Fig. 1E-F

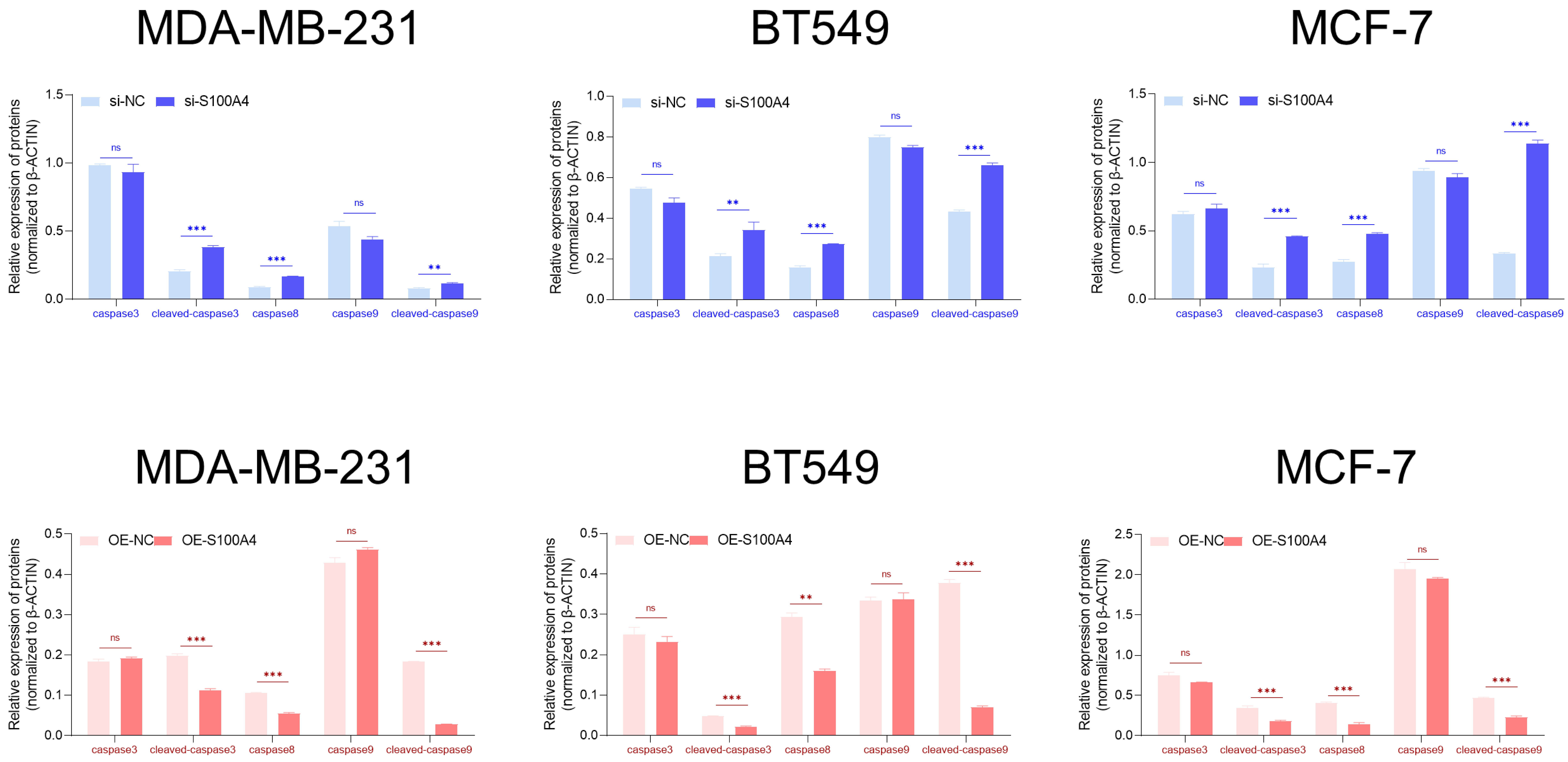

Western Blot analysis for Supplement Fig. 1G

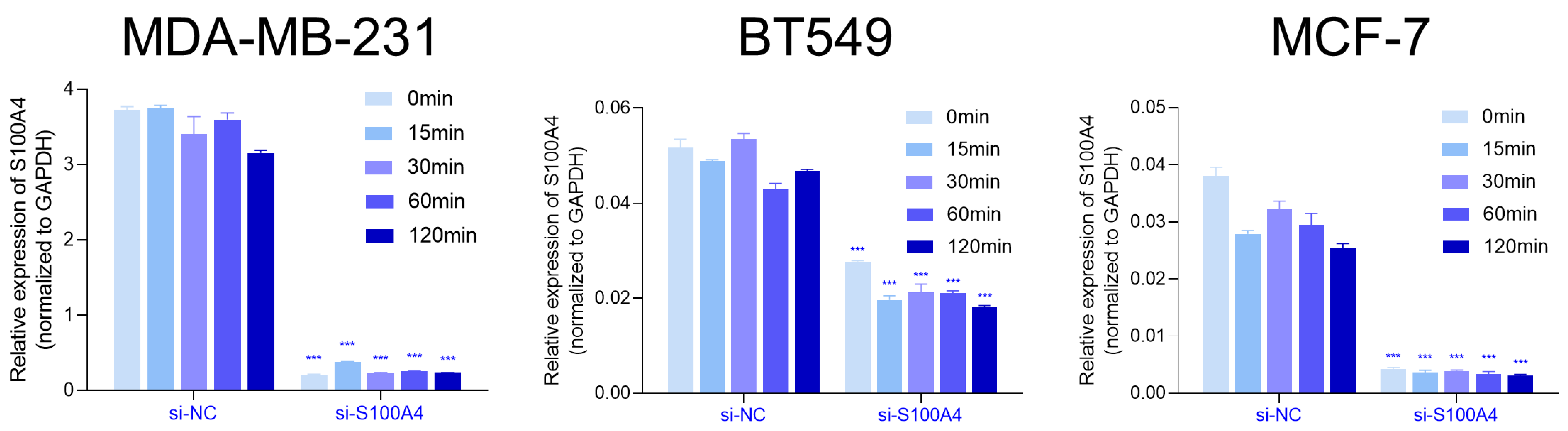

Supplement data 2. Sequences of primers, siRNAs and plasmids; details of primary and secondary antibodies.

1. The sequences of primers were listed below:

Name		Primer (5'-3')
β-actin	Forward	CCTCGCCTTTGCCGATCC
	Reverse	GGATCTTCATGAGGTAGTCAGTC
ANXA9	Forward	CAGCTCATCTCACGAAACTTCC
	Reverse	GGTTCGAGTGGCAAGAATTTCAA
S100A4	Forward	GATGAGCAACTTGGACAGCAA
	Reverse	CTGGGCTGCTTATCTGGGAAG

2. The sequences of siRNAs and details of vectors were listed below:

Name		Sequence (5'-3')
siRNA-negative control	Sense	UUCUCCGAACGUGUCACGUTT
	Antisense	ACGUGAACGUUCGGAGAATT
si-ANXA9-1	Sense	CCCAACAGGACCUGAUGAATT
	Antisense	UUCAUCAGGUCCUGUUGGGTT
si-ANXA9-2	Sense	GCAGUCUACAAACACAAUUTT
	Antisense	AAUUGUGUUUGUAGACUGCTT
si-S100A4	Sense	AAUUCGUUACACAUCAUGGCG
	Antisense	CCAUGAUGUGUAACGAAUUCU

- The vectors of over-expression plasmids were pCDH-CMV-MCS-EF1-copGFP-T2A-Puro (Generay Biotech, Shanghai, China)
- the vectors of shRNAs were PLKO.1 (GentleGen, Suzhou, China).

3. The primary and secondary antibodies involved were listed as followings:

Name	Dilution ratio	Cat#	Company
ANXA9	1:1000	ab166621	Abcam, Cambridge, UK
S100A4	1:1000	ab197896	Abcam, Cambridge, UK
Caspase3 and cleaved-caspase3	1:1000	T40044	Abmart, Shanghai, China
Caspase8	1:1000	T40045	Abmart, Shanghai, China
Caspase9 and cleaved-caspase9	1:1000	T40046	Abmart, Shanghai, China
p53	1:5000	10442-1-AP	Proteintech, Wuhan, China
Bcl-2	1:500	T40056	Abmart, Shanghai, China
Bax	1:500	A0207	Abclonal, Wuhan, China
AKT	1:1000	ab8805	Abcam, Cambridge, UK
p-AKT(S473)	1:500	AP0140	Abclonal, Wuhan, China
m-TOR	1:1000	T55306	Abmart, Shanghai, China
STAT3	1:1000	1122-1	epitomics, California, USA
p-STAT3(Y705)	1:1000	2236-1	epitomics, California, USA
HA-tag	1:2000	AE008	Abclonal, Wuhan, China
pan Phospho-Serine/Threonine	1:500	T91067	Abmart, Shanghai, China
β-ACTIN	1:2000	GB15003	Servicebio, Wuhan, China
GAPDH	1:2000	GB11002	Servicebio, Wuhan, China
Histone H3	1:500	A2348	Abclonal, Wuhan, China
ATP1A1	1:1000	GB11400-100	Servicebio, Wuhan, China
Goat Anti-Rabbit IgG (Dylight 800)	1:2000	A23920	abbkine, California, USA
Goat Anti-Mouse IgG (Dylight 800)	1:2000	A23910	abbkine, California, USA

The antibodies applied in IHC staining are listed as follows:

Name	Dilution ratio	Cat#	Company
p53	1:200	GB111740	Servicebio, Wuhan, China
Ki-67	1:300	GB121141	Servicebio, Wuhan, China
VEGF	1:200	GB14165	Servicebio, Wuhan, China
