# Supplementary material for: ANXA9 facilitates S100A4 and promotes breast cancer progression through modulating STAT3 pathway
Source: Cell Death Dis. 2024 Apr 12;15(4):260. doi: 10.1038/s41419-024-06643-4 (PMC11014919; doi:10.1038/s41419-024-06643-4)
Supplement: Supplementary file 3 — Supplement information [file 41419_2024_6643_MOESM3_ESM.pdf]

Supplement figure 1 Biological functions of S100A4 in breast cancer cells.

(A-B) S100A4 affected BC cellular proliferation abilities via MTT assays; (C-D) S100A4 affected BC cellular colony-forming ability; (E-F) S100A4 affected the protein expression of cleaved-caspase3 and cleaved-caspase9 in BC cells; (G-I) Si-S100A4 treatment weakened the time-dependent ubiquitin-proteasome degradation of p53 in BC cells in response to CHX treatment and MG-132 treatment (relative gray values); (J-K) sh-S100A4 inhibits the tumorigenic ability of MDA-MB-231 cells in nude mice; (L) IHC staining revealed that sh-S100A4 decreased Ki67 and increased p53 expression in xenograft tumors; (M) The sh-S100A4 group decreased the number of pulmonary metastatic foci in mice lung metastasis model. $^{*}p<0.05$, $^{**}p<0.01$, $^{***}p<0.001$. All of the experiments were replicated for three times.

Supplement table 1. Differentially expressed genes in Metastatic Breast Cancer.

Supplement data 2. Sequences of primers, siRNAs and plasmids; details of primary and secondary antibodies. （1）The sequences of primers. （2）The sequences of siRNAs and details of vectors. （3） The primary and secondary antibodies involved in experiments.

Supplement data 3. Gray scale analysis for western blots.

Gray scale analysis for western blots from figure 4G-I, 4J-L, 5K-M, 6A, 6B, 6C, 6D, 6E, 6F, 6G, 6H, 6I, 6J, 7D, 7E, 7F and 7G. And Gray scale analysis for western blots from supplement figure 1E-F and 1G.
