# Supplementary material for: ANXA9 facilitates S100A4 and promotes breast cancer progression through modulating STAT3 pathway
Source: Cell Death Dis. 2024 Apr 12;15(4):260. doi: 10.1038/s41419-024-06643-4 (PMC11014919; doi:10.1038/s41419-024-06643-4)
Supplement: Supplementary file 4 — Original Data File [file 41419_2024_6643_MOESM4_ESM.pdf]

Original blots of western blot

Figure 1C

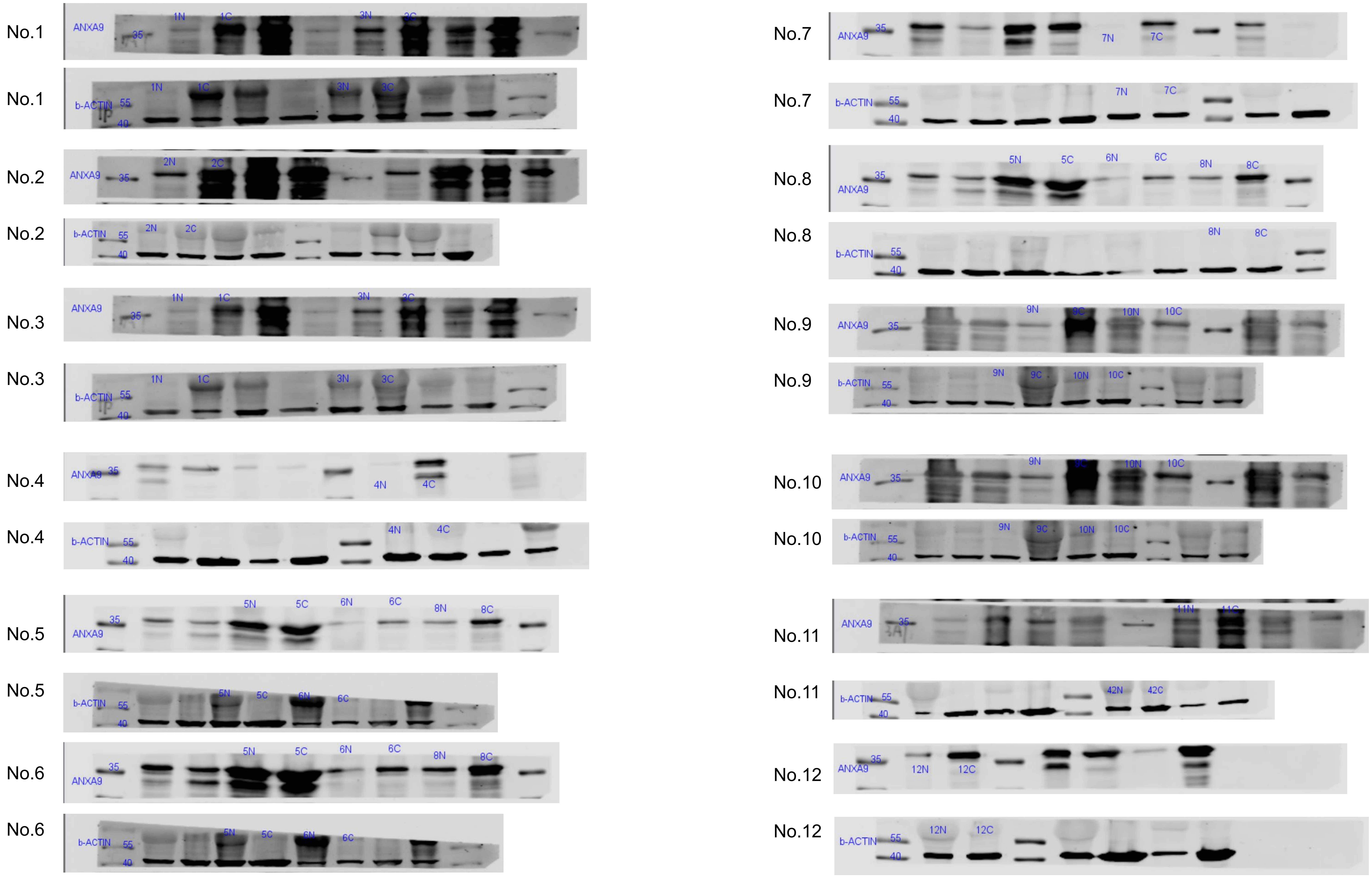
No.1
ANXA9
b-ACTIN
No.2
No.3
No.4
No.5
No.6
No.7
No.8
No.9
No.10
No.11
No.12

Figure 4D-F

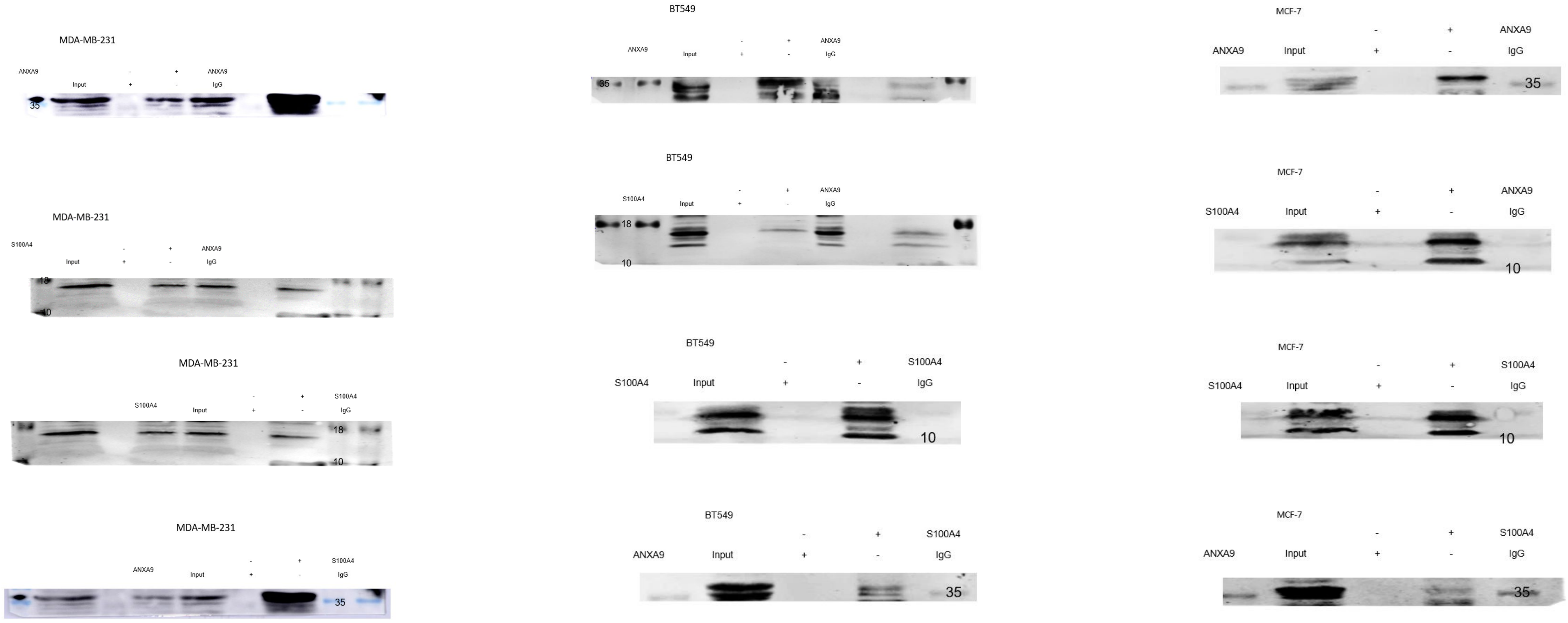

Figure 4G-I

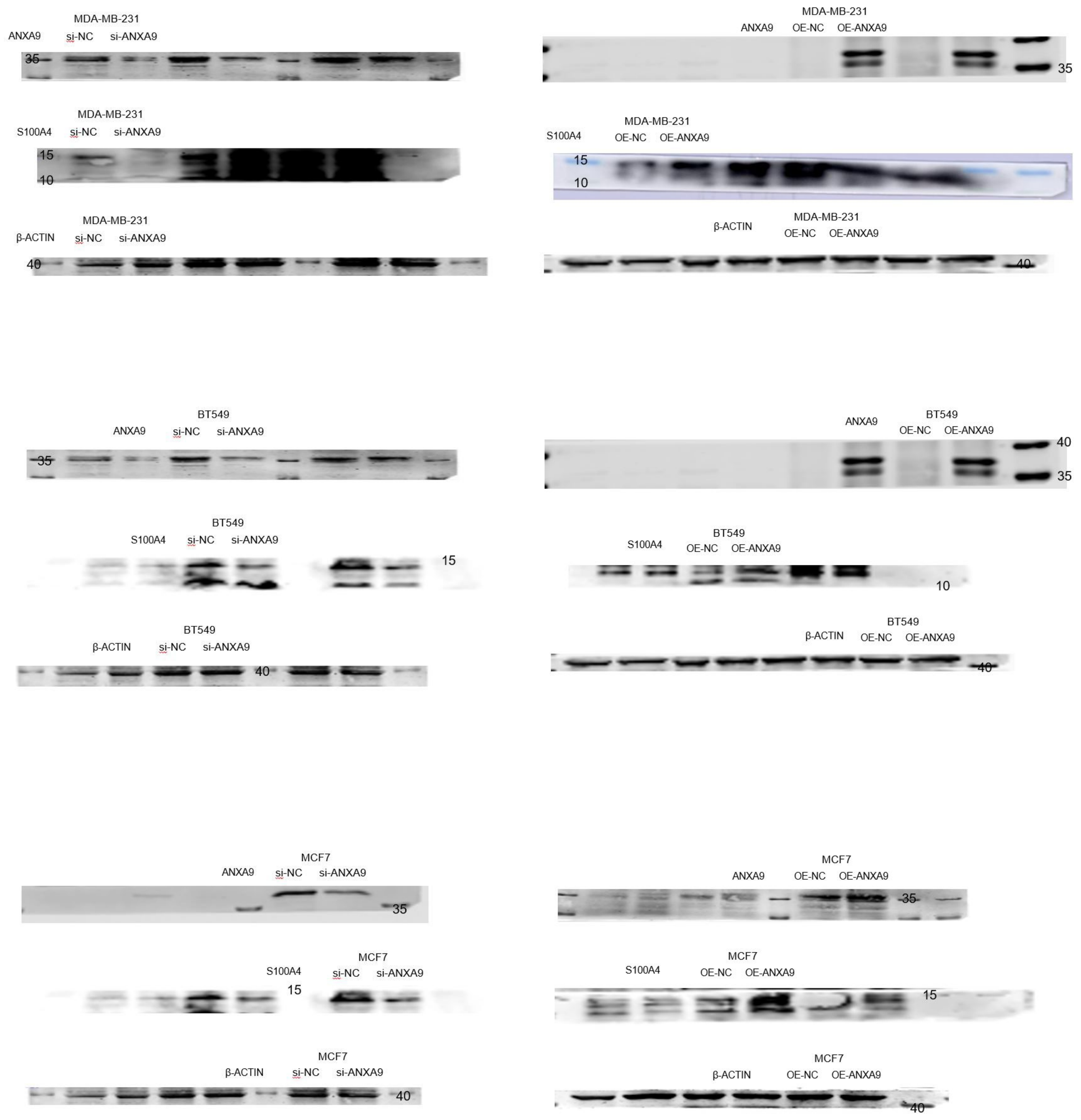

MDA-MB-231
ANXA9
si-NC
si-ANXA9
35
S100A4
15
10
β-ACTIN
40
OE-NC
OE-ANXA9
BT549
MCF7

Figure 4J-L

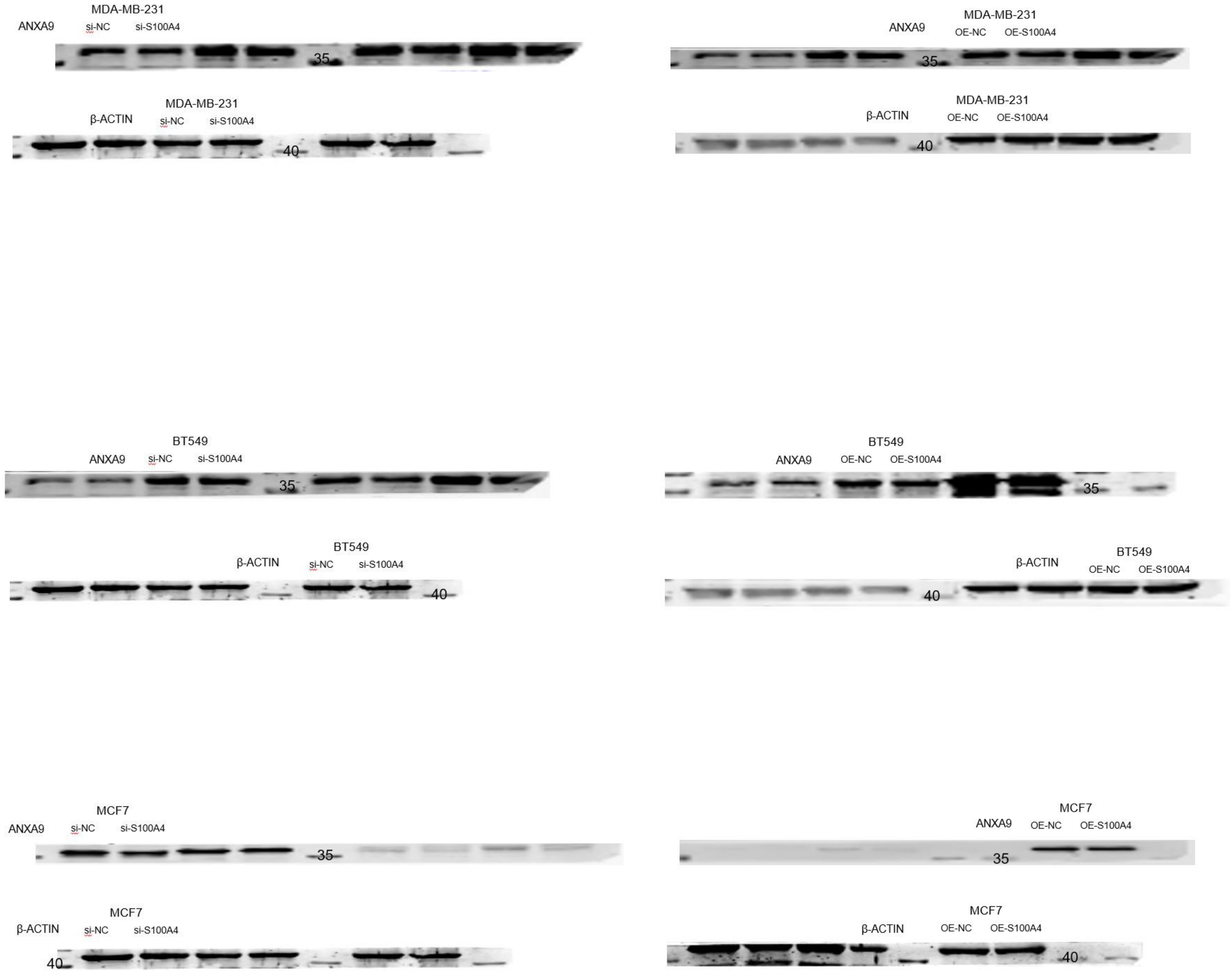
ANXA9
MDA-MB-231
si-NC
si-S100A4
35
β-ACTIN
MDA-MB-231
si-NC
si-S100A4
40
ANXA9
MDA-MB-231
OE-NC
OE-S100A4
35
β-ACTIN
MDA-MB-231
OE-NC
OE-S100A4
40
ANXA9
BT549
si-NC
si-S100A4
35
β-ACTIN
BT549
si-NC
si-S100A4
40
ANXA9
BT549
OE-NC
OE-S100A4
35
β-ACTIN
BT549
OE-NC
OE-S100A4
40
ANXA9
MCF7
si-NC
si-S100A4
35
β-ACTIN
MCF7
si-NC
si-S100A4
40
ANXA9
MCF7
OE-NC
OE-S100A4
35
β-ACTIN
MCF7
OE-NC
OE-S100A4
40

Figure 5A-C

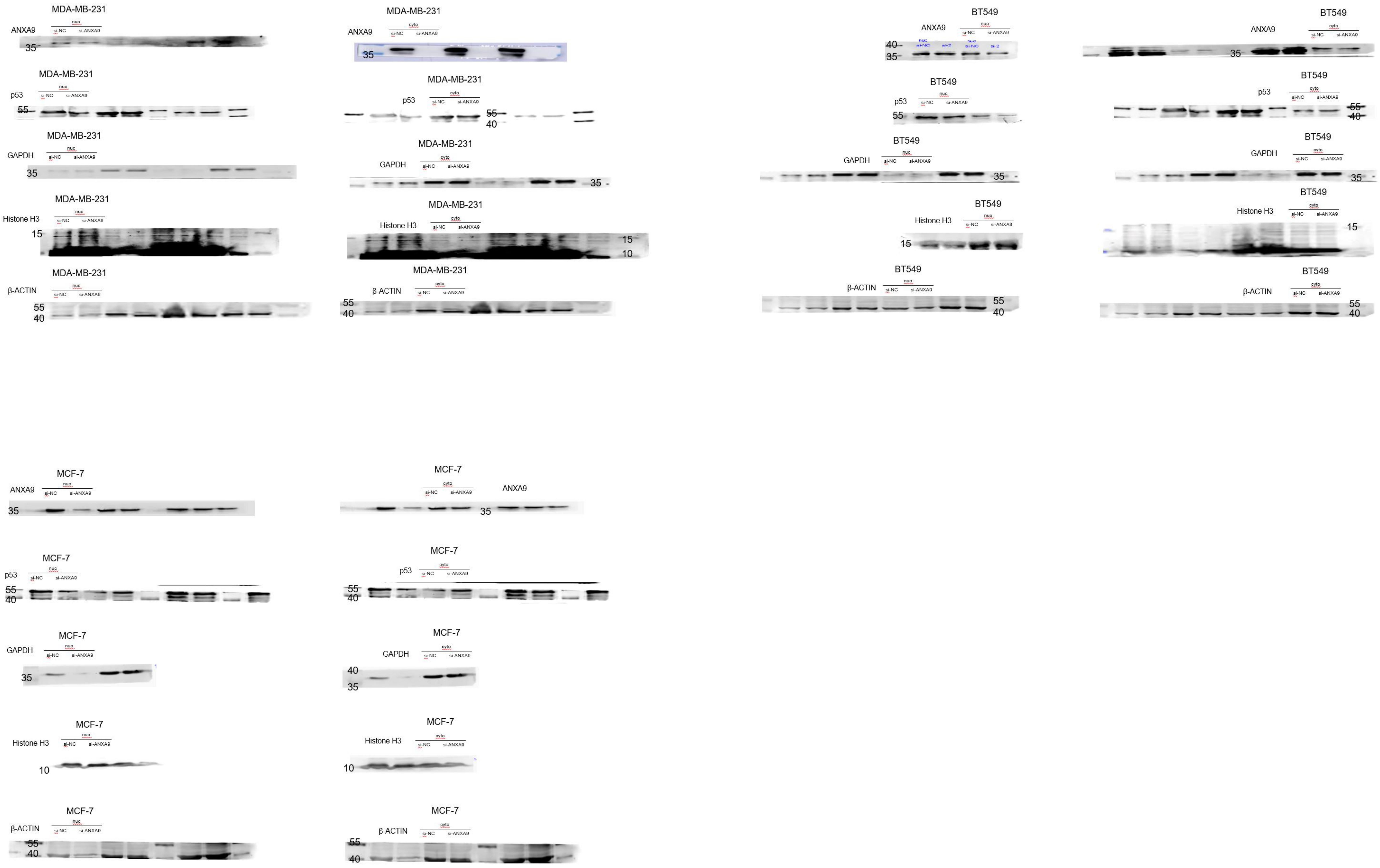
MDA-MB-231
ANXA9
nuc.
si-NC
si-ANXA9
cyto
35
p53
55
40
GAPDH
Histone H3
15
10
β-ACTIN
BT549
MCF-7

Figure 5F-H

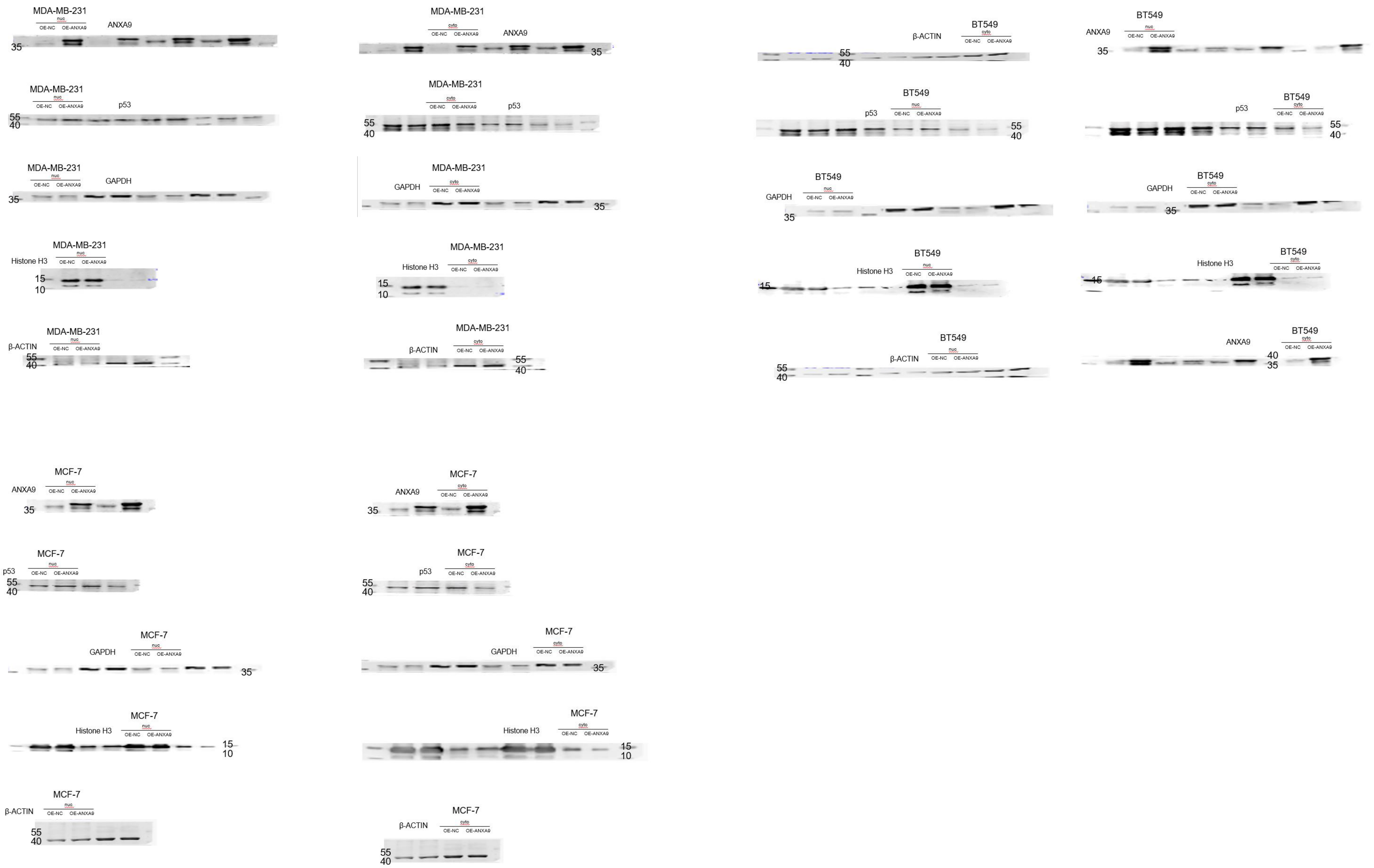
MDA-MB-231
nuc
OE-NC
OE-ANXA9
ANXA9
35
MDA-MB-231
cyto
OE-NC
OE-ANXA9
ANXA9
35
p53
55
40
GAPDH
35
Histone H3
15
10
β-ACTIN
55
40
BT549
ANXA9
35
β-ACTIN
p53
GAPDH
Histone H3
15
MCF-7
ANXA9
35
p53
55
40
GAPDH
35
Histone H3
15
10
β-ACTIN
55
40

Figure 6A-B

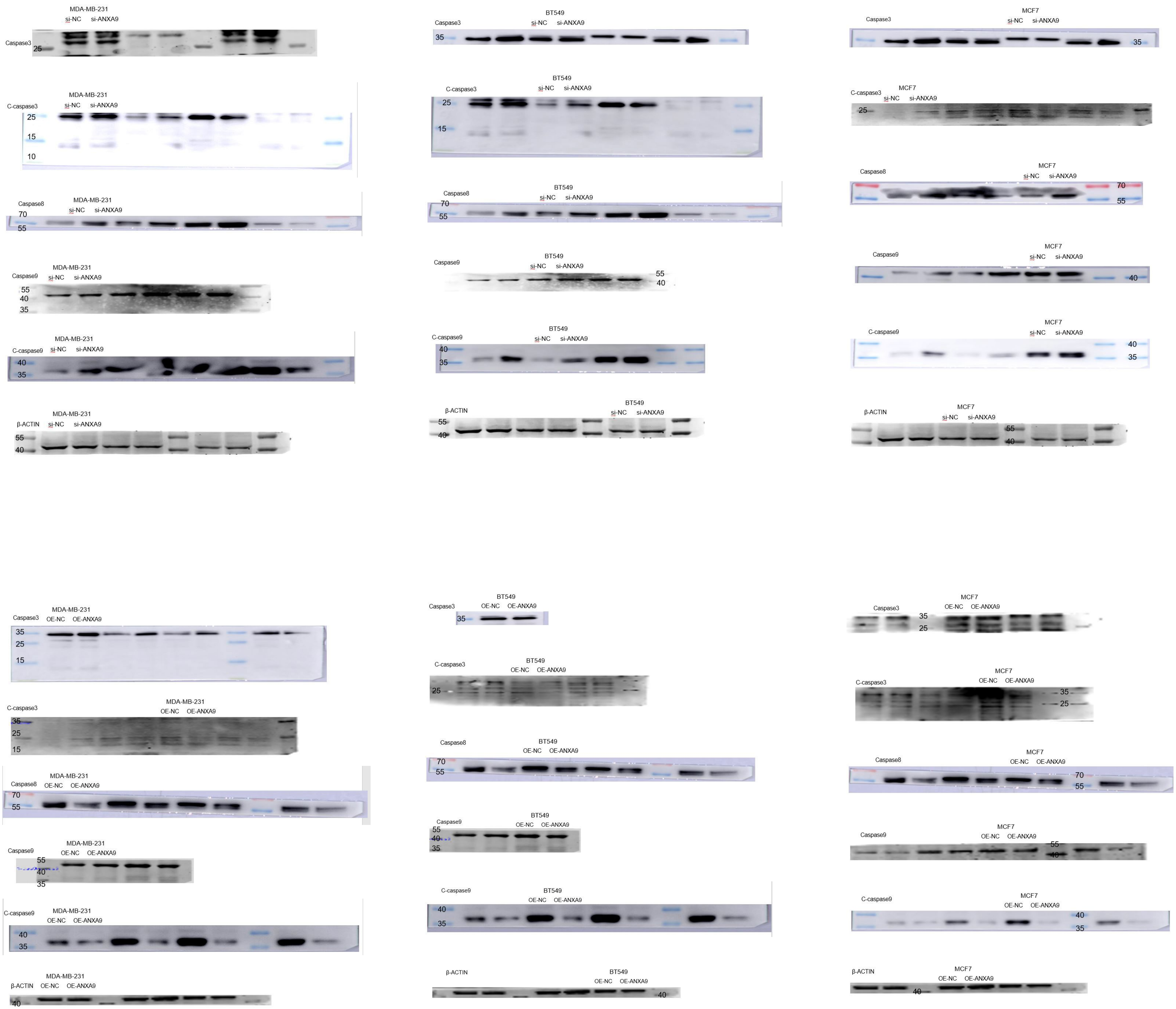
MDA-MB-231
si-NC si-ANXA9
Caspase3
25
C-caspase3
25
15
10
Caspase8
70
55
Caspase9
55
40
35
C-caspase9
40
35
β-ACTIN
55
40
BT549
si-NC si-ANXA9
Caspase3
35
C-caspase3
25
15
Caspase8
70
55
Caspase9
55
40
C-caspase9
40
35
β-ACTIN
55
40
MCF7
si-NC si-ANXA9
Caspase3
35
C-caspase3
25
Caspase8
70
55
Caspase9
40
C-caspase9
40
35
β-ACTIN
55
40
MDA-MB-231
OE-NC OE-ANXA9
Caspase3
35
25
15
C-caspase3
35
25
15
Caspase8
70
55
Caspase9
55
40
35
C-caspase9
40
35
β-ACTIN
40
BT549
OE-NC OE-ANXA9
Caspase3
35
C-caspase3
25
Caspase8
70
55
Caspase9
55
40
35
C-caspase9
40
35
β-ACTIN
40
MCF7
OE-NC OE-ANXA9
Caspase3
35
25
C-caspase3
35
25
Caspase8
70
55
Caspase9
55
40
C-caspase9
40
35
β-ACTIN
40

Figure 6C-D

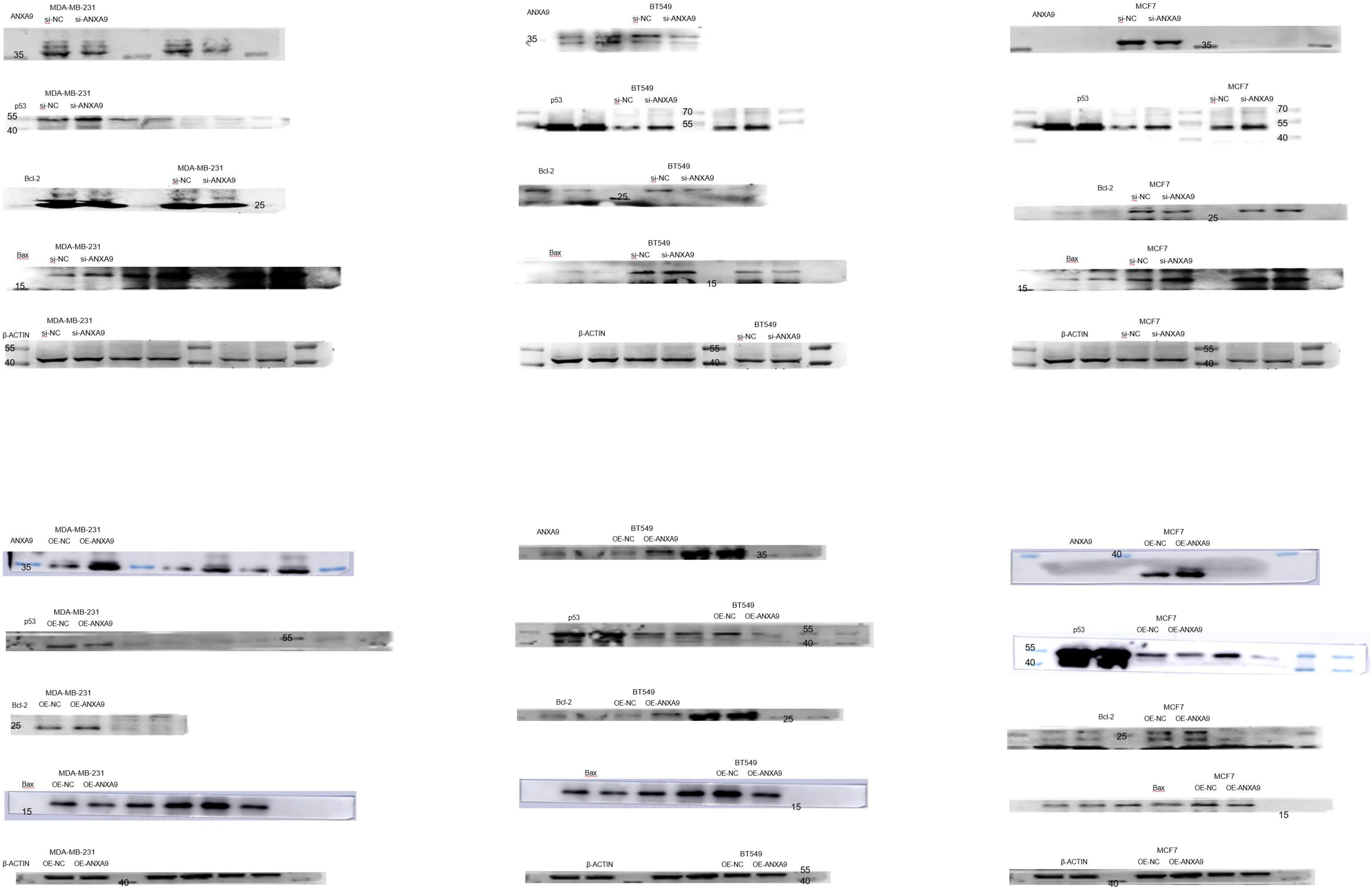
ANXA9
MDA-MB-231
si-NC si-ANXA9
35
ANXA9
BT549
si-NC si-ANXA9
35
ANXA9
MCF7
si-NC si-ANXA9
35
p53
MDA-MB-231
si-NC si-ANXA9
55
40
p53
BT549
si-NC si-ANXA9
70
55
p53
MCF7
si-NC si-ANXA9
70
55
40
Bcl-2
MDA-MB-231
si-NC si-ANXA9
25
Bcl-2
BT549
si-NC si-ANXA9
25
Bcl-2
MCF7
si-NC si-ANXA9
25
Bax
MDA-MB-231
si-NC si-ANXA9
15
Bax
BT549
si-NC si-ANXA9
15
Bax
MCF7
si-NC si-ANXA9
15
β-ACTIN
MDA-MB-231
si-NC si-ANXA9
55
40
β-ACTIN
BT549
si-NC si-ANXA9
55
40
β-ACTIN
MCF7
si-NC si-ANXA9
55
40
ANXA9
MDA-MB-231
OE-NC OE-ANXA9
35
ANXA9
BT549
OE-NC OE-ANXA9
35
ANXA9
MCF7
OE-NC OE-ANXA9
40
p53
MDA-MB-231
OE-NC OE-ANXA9
55
p53
BT549
OE-NC OE-ANXA9
55
40
p53
MCF7
OE-NC OE-ANXA9
55
40
Bcl-2
MDA-MB-231
OE-NC OE-ANXA9
25
Bcl-2
BT549
OE-NC OE-ANXA9
25
Bcl-2
MCF7
OE-NC OE-ANXA9
25
Bax
MDA-MB-231
OE-NC OE-ANXA9
15
Bax
BT549
OE-NC OE-ANXA9
15
Bax
MCF7
OE-NC OE-ANXA9
15
β-ACTIN
MDA-MB-231
OE-NC OE-ANXA9
40
β-ACTIN
BT549
OE-NC OE-ANXA9
55
40
β-ACTIN
MCF7
OE-NC OE-ANXA9
40

Figure 6E-F

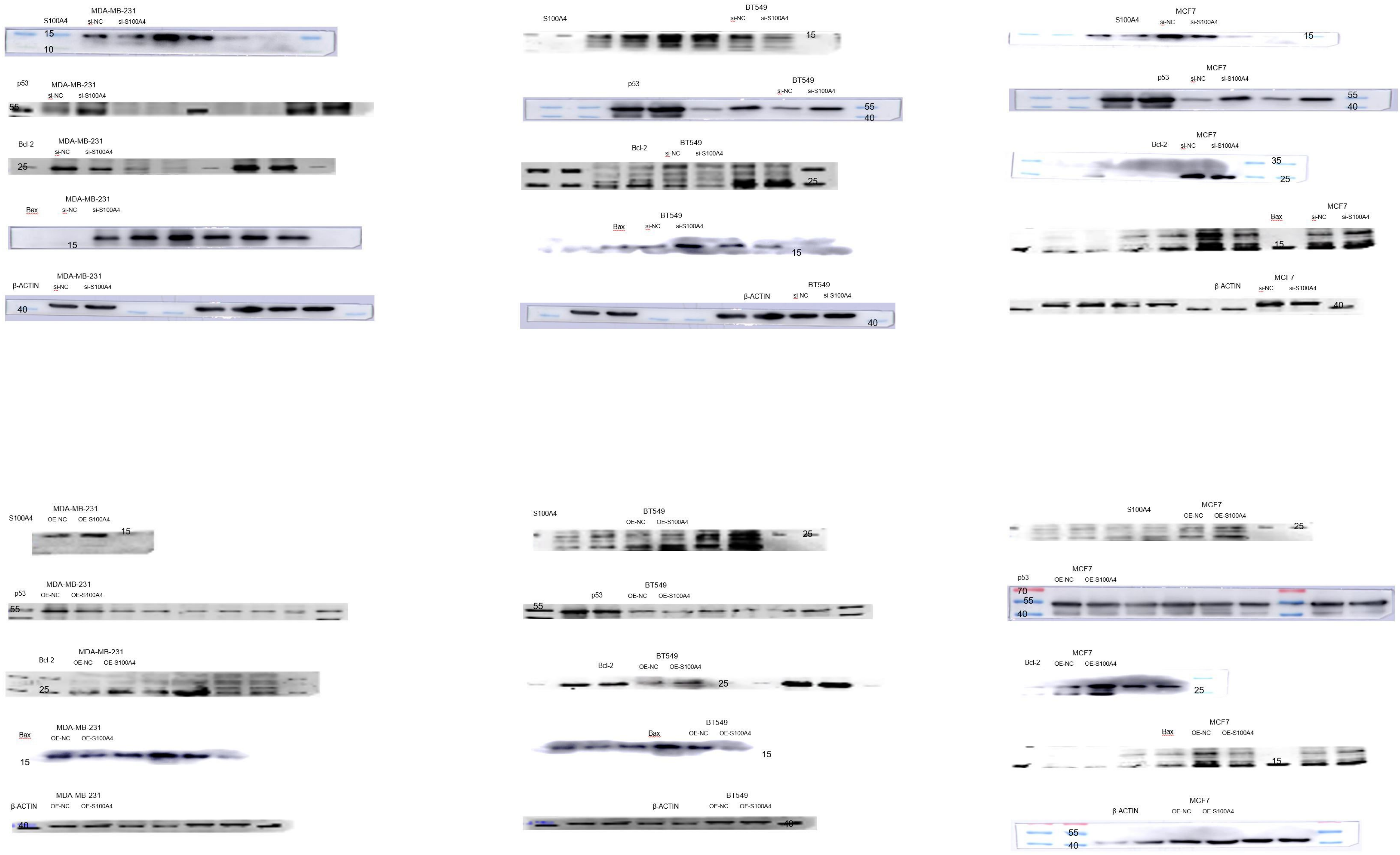
MDA-MB-231
S100A4
si-NC
si-S100A4
p53
Bcl-2
Bax
β-ACTIN
BT549
MCF7
OE-NC
OE-S100A4
15
10
55
40
25
35
70

Figure 6G-H

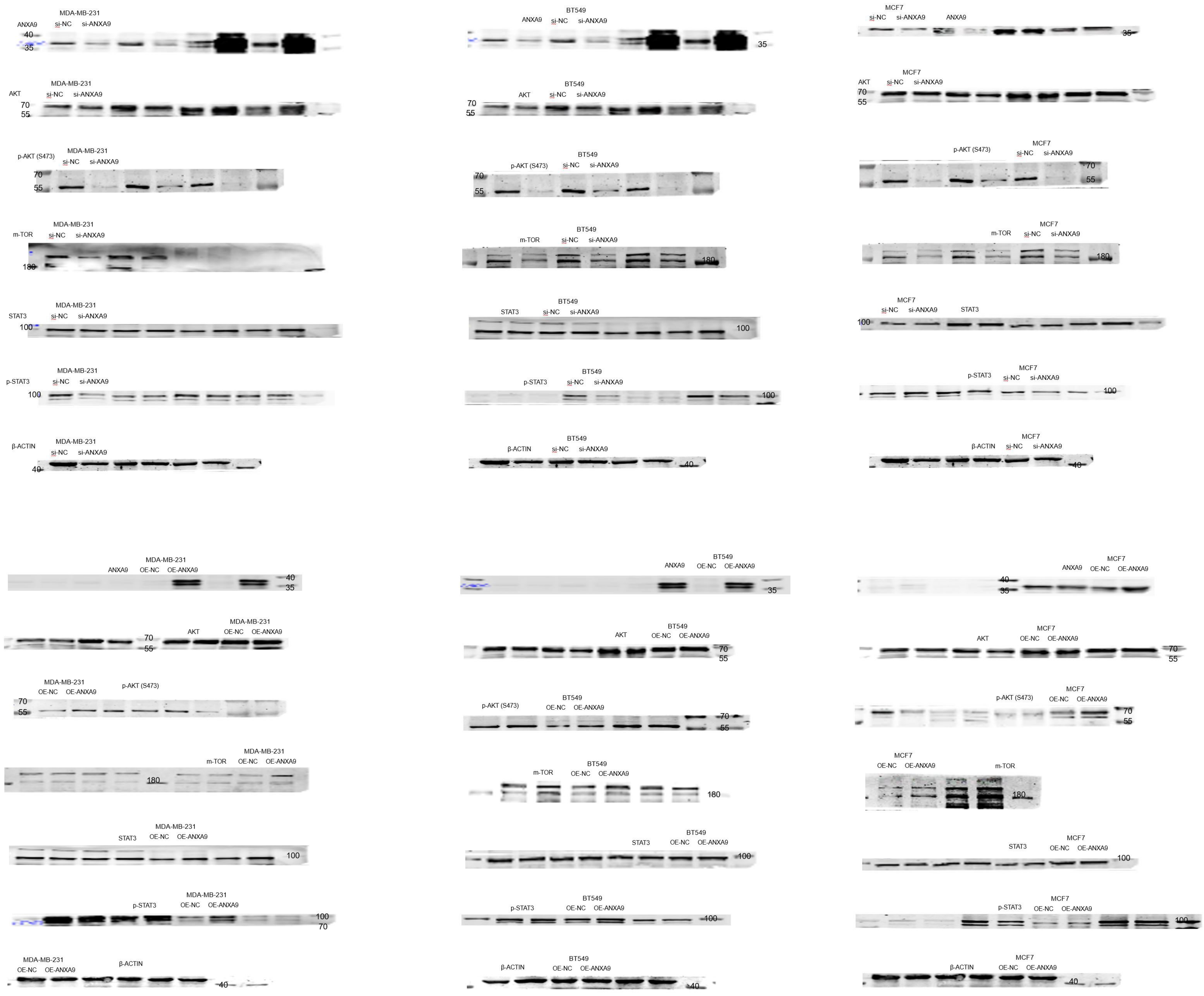

MDA-MB-231
ANXA9
si-NC
si-ANXA9
40
35
BT549
MCF7
AKT
70
55
p-AKT (S473)
m-TOR
180
STAT3
100
p-STAT3
β-ACTIN
OE-NC
OE-ANXA9

Figure 6I-J

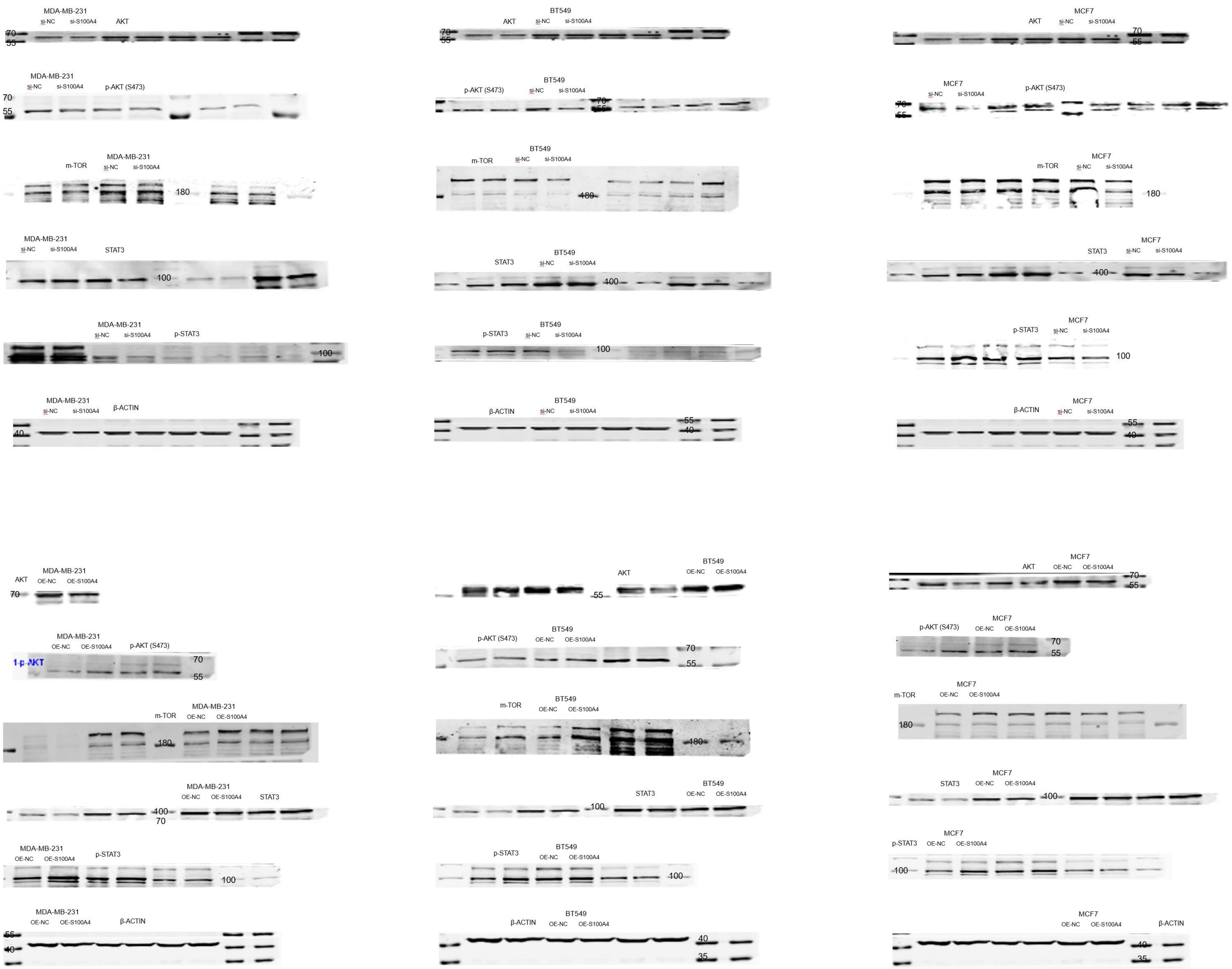
MDA-MB-231
si-NC
si-S100A4
AKT
p-AKT (S473)
m-TOR
STAT3
p-STAT3
β-ACTIN
BT549
MCF7
OE-NC
OE-S100A4
70
55
180
100
40
35
1 p-AKT

Figure 7D-E

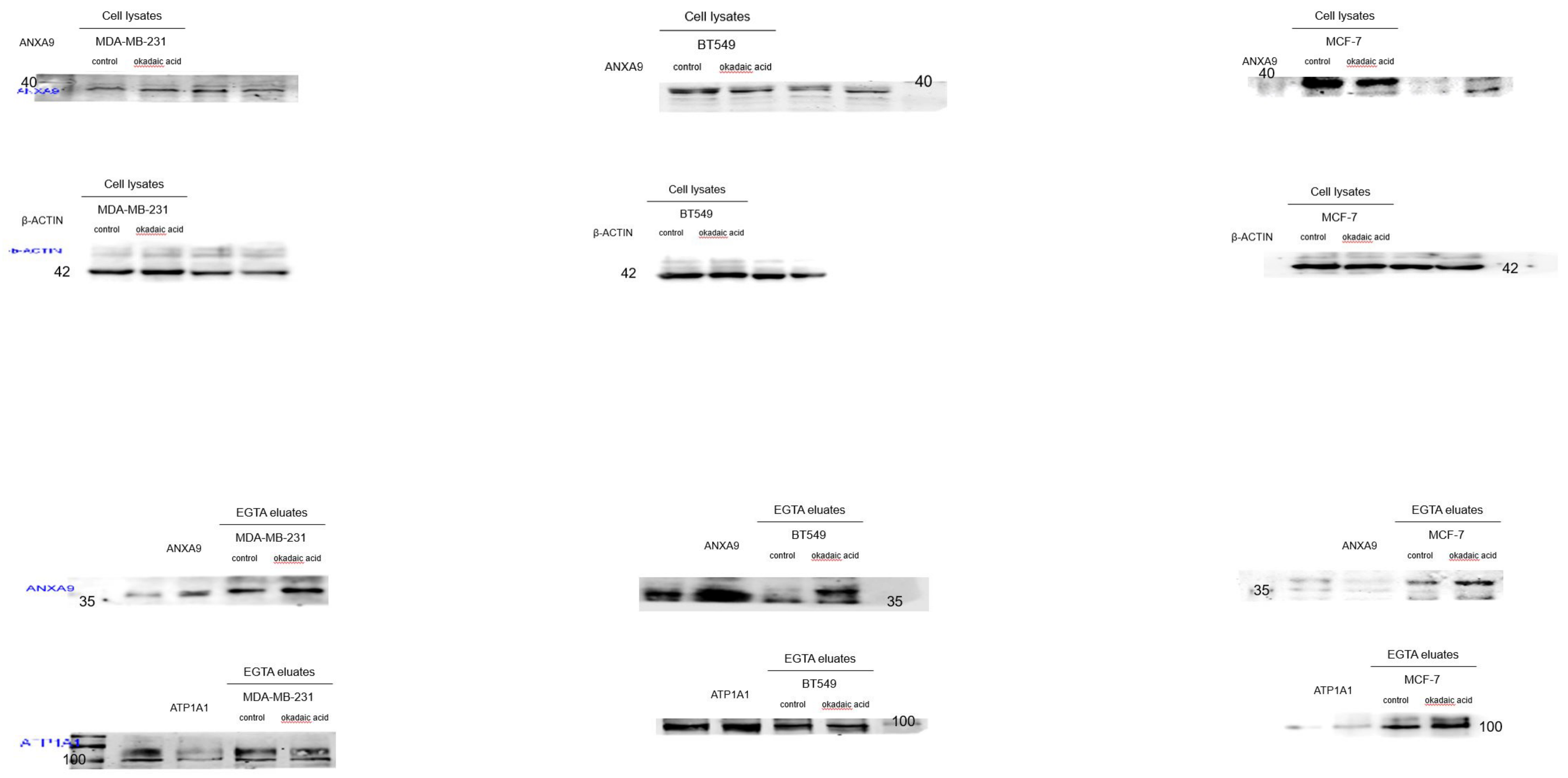

Figure 7F

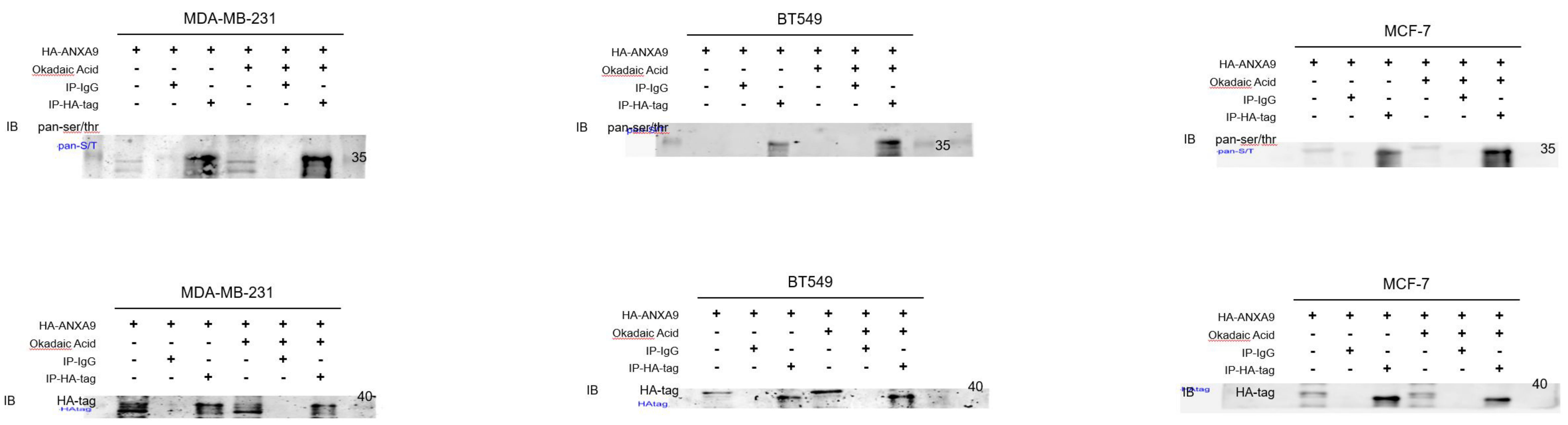

Figure 7G

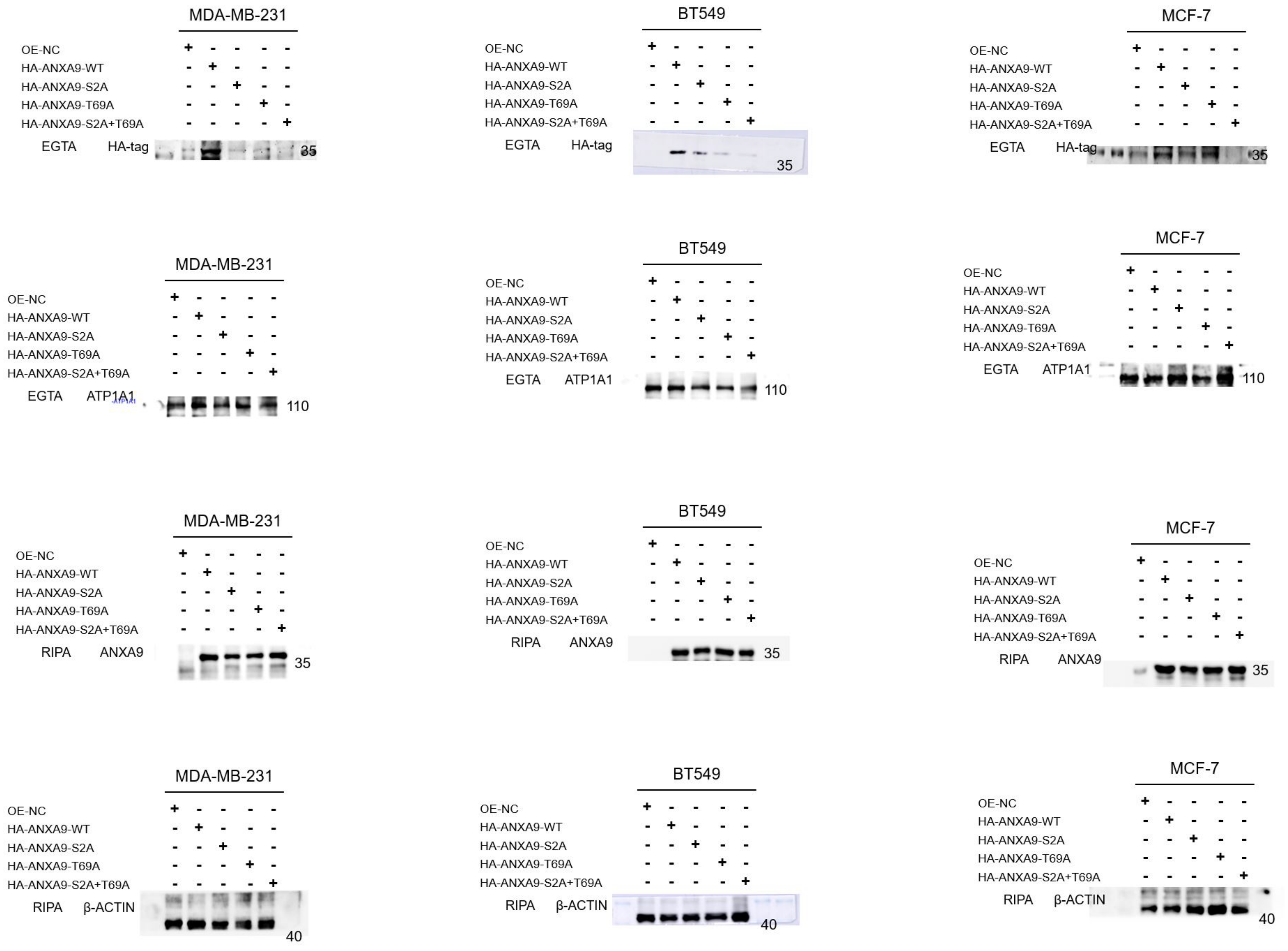
MDA-MB-231
OE-NC
HA-ANXA9-WT
HA-ANXA9-S2A
HA-ANXA9-T69A
HA-ANXA9-S2A+T69A
EGTA
HA-tag
35
BT549
OE-NC
HA-ANXA9-WT
HA-ANXA9-S2A
HA-ANXA9-T69A
HA-ANXA9-S2A+T69A
EGTA
HA-tag
35
MCF-7
OE-NC
HA-ANXA9-WT
HA-ANXA9-S2A
HA-ANXA9-T69A
HA-ANXA9-S2A+T69A
EGTA
HA-tag
35
MDA-MB-231
OE-NC
HA-ANXA9-WT
HA-ANXA9-S2A
HA-ANXA9-T69A
HA-ANXA9-S2A+T69A
EGTA
ATP1A1
110
BT549
OE-NC
HA-ANXA9-WT
HA-ANXA9-S2A
HA-ANXA9-T69A
HA-ANXA9-S2A+T69A
EGTA
ATP1A1
110
MCF-7
OE-NC
HA-ANXA9-WT
HA-ANXA9-S2A
HA-ANXA9-T69A
HA-ANXA9-S2A+T69A
EGTA
ATP1A1
110
MDA-MB-231
OE-NC
HA-ANXA9-WT
HA-ANXA9-S2A
HA-ANXA9-T69A
HA-ANXA9-S2A+T69A
RIPA
ANXA9
35
BT549
OE-NC
HA-ANXA9-WT
HA-ANXA9-S2A
HA-ANXA9-T69A
HA-ANXA9-S2A+T69A
RIPA
ANXA9
35
MCF-7
OE-NC
HA-ANXA9-WT
HA-ANXA9-S2A
HA-ANXA9-T69A
HA-ANXA9-S2A+T69A
RIPA
ANXA9
35
MDA-MB-231
OE-NC
HA-ANXA9-WT
HA-ANXA9-S2A
HA-ANXA9-T69A
HA-ANXA9-S2A+T69A
RIPA
β-ACTIN
40
BT549
OE-NC
HA-ANXA9-WT
HA-ANXA9-S2A
HA-ANXA9-T69A
HA-ANXA9-S2A+T69A
RIPA
β-ACTIN
40
MCF-7
OE-NC
HA-ANXA9-WT
HA-ANXA9-S2A
HA-ANXA9-T69A
HA-ANXA9-S2A+T69A
RIPA
β-ACTIN
40
